# Application of Nano-Inspired Scaffolds-Based Biopolymer Hydrogel for Bone and Periodontal Tissue Regeneration

**DOI:** 10.3390/polym14183791

**Published:** 2022-09-10

**Authors:** Sheikha A. Alkhursani, Mohamed Mohamady Ghobashy, Samera Ali Al-Gahtany, Abeer S. Meganid, Shady M. Abd El-Halim, Zubair Ahmad, Farhat S. Khan, Gamal Abdel Nasser Atia, Simona Cavalu

**Affiliations:** 1 Faculty of Science and Humanities-Jubail, Imam Abdulrahman Bin Faisal University, Jubail 31441, Saudi Arabia; 2 Radiation Research of Polymer Chemistry Department, National Center for Radiation Research and Technology (NCRRT), Atomic Energy Authority, Cairo 11787, Egypt; 3 Faculty of Science, University of Jeddah, Jeddah 21959, Saudi Arabia; 4 Department of Pharmaceutics and Industrial Pharmacy, Faculty of Pharmacy, October 6 University, 6th of October City, Giza 12585, Egypt; 5 Unit of Bee Research and Honey Production, Faculty of Science, King Khalid University, Abha 61413, Saudi Arabia; 6 Biology Department, College of Arts and Sciences, Dehran Al-Junub, King Khalid University, Abha 61413, Saudi Arabia; 7 Department of Oral Medicine, Periodontology and Diagnosis, Faculty of Dentistry, Suez Canal University, Ismailia 41522, Egypt; 8 Faculty of Medicine and Pharmacy, University of Oradea, P-ta 1 Decembrie 10, 410087 Oradea, Romania

**Keywords:** polysaccharide, protein, collagen, bone generation, periodontal, nanoscale

## Abstract

This review’s objectives are to provide an overview of the various kinds of biopolymer hydrogels that are currently used for bone tissue and periodontal tissue regeneration, to list the advantages and disadvantages of using them, to assess how well they might be used for nanoscale fabrication and biofunctionalization, and to describe their production processes and processes for functionalization with active biomolecules. They are applied in conjunction with other materials (such as microparticles (MPs) and nanoparticles (NPs)) and other novel techniques to replicate physiological bone generation more faithfully. Enhancing the biocompatibility of hydrogels created from blends of natural and synthetic biopolymers can result in the creation of the best scaffold match to the extracellular matrix (ECM) for bone and periodontal tissue regeneration. Additionally, adding various nanoparticles can increase the scaffold hydrogel stability and provide a number of biological effects. In this review, the research study of polysaccharide hydrogel as a scaffold will be critical in creating valuable materials for effective bone tissue regeneration, with a future impact predicted in repairing bone defects.

## 1. Introduction

Polysaccharide hydrogel has recently emerged with the potential to revolutionize the bone regeneration field [1]. The medical procedure of nanoscaffolding polysaccharide is used to regenerate a bone and tissue, including organs and limbs. The nanoscaffold is a 3D structure made of tiny polysaccharide hydrogel fibers scaled down from the nanometer scale (10^−9^ m) [2]. Because of their distinct chemical and physical characteristics (e.g., magnetic and electrical), nanostructured biomaterials have outperformed their bulk counterparts in terms of improving bone regeneration [3]. Moreover, compared with bulk materials, nanostructured biomaterials can be designed to match the bone’s composition and nanoarchitecture and recapitulate the key properties of its biochemical milieu at the nanoscale. These characteristics lead to an enhanced engagement between the host immune system and cells of a progenitor at the nanometer size, in turn yielding better results.

The treatment of bone abnormalities is still a major issue in the orthopedic sector, and much research is being conducted to develop appropriate treatments [4]. For maxillofacial and periodontal surgeons, alveolar bone repair and augmentation are challenging and demanding. The major goal of these procedures is to restore bone density in patients who have lost it due to various factors, such as periodontal disease, age, reconstructive surgery, neoplastic pathology, congenital abnormalities, osteoporosis, and trauma [5]. 

The periodontium is the connecting tissue that supports teeth. It consists of the cementum (CM), alveolar bone (AB), periodontal ligament (PDL), and gingiva, a tissue unit that surrounds and supports the teeth. Periodontitis is an inflammatory reaction induced by plaque microorganisms that destroy the periodontium. It is one of the most frequent illnesses seen in dental offices. Periodontitis is the prevalent cause of the loosening of a tooth, movement, and even loss, which, in turn, significantly impact mastication, food intake, and aesthetics [6]. Initial periodontal therapy, surgery of the periodontal flap, and regeneration of bone tissue are all treatment methods for periodontitis that can lower the depth of the periodontal pocket probing and partially repair periodontal attachment, but their results are still limited and need further investigation [7]. As existing therapies primarily focus on either anti-inflammation of the tissue or the regeneration of the periodontium tissue, they cannot heal the damaged periodontium entirely [8]. Thus, exploring a unique approach to suppress inflammation while also promoting the regeneration of injured periodontal tissue is still of tremendous scientific significance. From an anatomical and physiological standpoint, the functional integration of scaffolds and/or matrices that simultaneously direct the regeneration of soft tissues and hard tissues is challenging [9]. Third-generation biomaterials and sophisticated processing methods have permitted a shift in the existing production approach, resulting in scaffolds of the bone cell with customized characteristics for demanding applications, such as functional and load-active compounds, such as protein or drugs [10]. Active medical compounds are integrated with additive biofunctional materials, enabling and enhancing the manufacturing of highly customized medical equipment of bone regeneration applications and bone implants [11]. These biofunctional materials for bone defect treatment can be hybrid and combine in situ with polysaccharide hydrogel, which is in high demand [12]. Additive biomanufacturing refers to the additive manufacturing technology translation into the tissue engineering of bone defect therapy and provides considerable advantages in the periodontal regeneration [13]. Advanced materials are critical for transitioning tissue engineering technologies from bench to bedside [14]. 

Any biomaterial, biologic or synthetic, intended for implantation in humans with the goal of restoring bone health, preserving bone structure, or filling bone loss is considered a bone substitute. The four main sources of accessible bone replacement materials are the patient himself (autogenous grafts), a different donor from the same species (allogeneic grafts), donors from a different species (xenogeneic grafts), and synthetically created materials (alloplastics). Each and every type of bone graft biomaterial has drawbacks, including those linked to the host response (immune reactions), amount, qualities following manufacturing processes, quick resorption, and others. The features of the material itself, the type of bone defect to be treated, the operator’s preferences, the associated expenses, and the patient’s acceptance are currently used to determine the best material for a given intervention. There are a wide variety of clinical conditions, so there may not be a single material that can be used to treat them all. Instead, it is crucial to pay attention to the material’s characteristics, as well as its formulation and presentation that may be best suited for each individual clinical condition. In terms of bone substitutes, autologous bone is still regarded as the gold standard [15]; however, clinical success is not always assured, and problems can happen in 8–39% of instances. The unpredictability of its resorption, the requirement for a second surgical surgery at the donor site, and the volume extracted that may not be adequate for some deformities are some major drawbacks of this type of graft. Allografts lack osteogenic qualities because they contain no live cells, but they do demonstrate osteoinductive and osteoconductive activity. Allogeneic and autogenous bone grafts both have their benefits and drawbacks, while xenogeneic bone grafts offer an alternative. Since they are neither osteoinductive nor osteogenic, these materials typically exhibit osteoconduction properties. According to certain articles, new xenografts might still fit within the traditional theory, or they might even exhibit osteoinductive qualities. Due to their resemblance to human bone in terms of chemical composition and structure, the majority of xenografts currently being employed are of swine and bovine origin. Since porcine-derived xenografts come from an animal species with a genotype similar to that of humans, they have undergone and continue to undergo extensive investigation to determine their potential as bone substitutes. The current focus is on developing synthetic polysaccharide hydrogel scaffolds capable of improving bone osteogenesis and bone vascularization in critical size defects [16]. Tissue engineering scaffolds are made of polysaccharide hydrogels. They provide polysaccharide-based cross-linked hydrogels with endless possibilities in drug delivery, notably in the transport of stem cells (SCs), different methodologies for structural alterations, and diversified polysaccharide-based cross-linked hydrogel creation with bioactive chemical composition, topography texturing, roughness surface, variation in size, connectivity, and shape of porosity [17]. In bone polysaccharide hydrogel scaffolds, all of these properties should be present and even must be bioresorbable at a pace consistent with the rate of production of a new bone for them to be completely replaced with a new bone cell [18]. Although the chemical composition and surface characteristics aid in osteogenesis and the formation of new bone cells, only the existence of interconnected porosity networks allows for widespread scaffold colonization and incorporation with new cells of the bone. These features can be found in polymeric materials, especially natural polysaccharides. Natural polysaccharides, including nanocellulose, alginate, and chitosan are abundant and can be mixed by triggering particular processes that regulate crucial physical–chemical interactions. In this regard, a recent study emphasized the importance of a conjugating Sr^2+^-doped apatitic cement with polysaccharide hydrogel scaffolds including alginate compounds to provide increased injectability using a surgical cannula and excellent osteointegrability and osteogenicity [19,20].

The present review focuses on natural polysaccharides since synthetic polymers have poor biocompatibility and bioactivity and a low number of cell adhesion sites. Natural polymers have been a popular choice for creating matrices that accurately replicate biological settings because of their resemblance to the components of the native natural extracellular matrix (ECM) in the human body. Their precise construction can result in a platform of sophisticated supporting materials, such as polysaccharide hydrogel scaffold with an adjustable fibrous and porous architecture structure. The elasticity and absorption of the polysaccharide hydrogel texture were coupled with the mechanical and osteoconductive characteristics of ceramics in composite ceramic–polysaccharide hydrogel scaffolds. Finally, the growth factor of bone cells, protein morphogenesis of bone cells, and bone cell osteogenesis were introduced to enhance the biological performance of a polysaccharide hydrogel scaffold. As a result, the hunt for appropriate materials and creative techniques to accurately replicate a bone microenvironment is ongoing, and various formulations are being created and evaluated to enhance their in vivo performance. In this study, we overview different tissue constructions based on polysaccharide hydrogel currently being modified for bone tissue engineering (BTE), including procedures of manufacturing processes of polysaccharide hydrogel.

**Figure 1:**
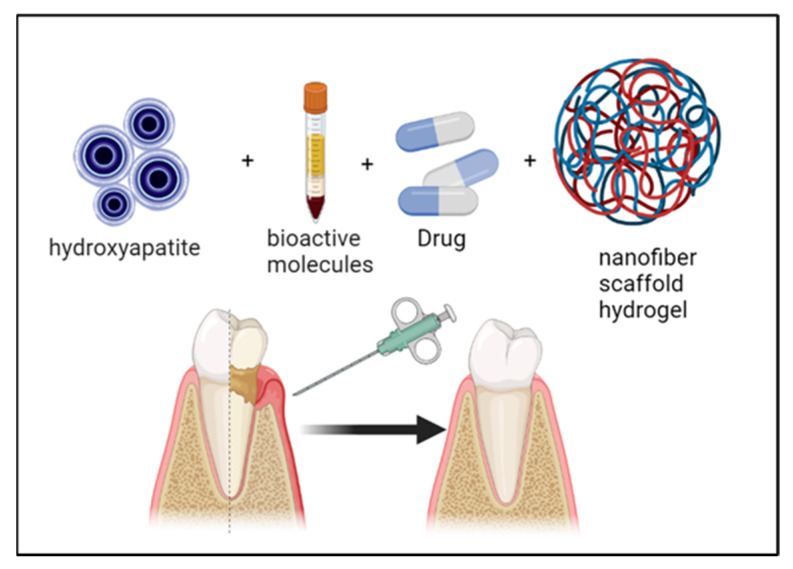
Hydrogel-based scaffolds for periodontal regeneration.

Biomaterial composition and scaffolding architecture are two important factors to consider when employing polysaccharide hydrogel scaffolds for periodontal regeneration. Furthermore, polysaccharide hydrogel scaffolds have several functions, such as controlling drug delivery, and can be carrying bioactive molecules (Figure 1). Injectable polysaccharide hydrogel can be applied to bony defects and cross-linked in situ; therefore, they are preferred for repairing irregular periodontal defects. However, the main downside of a scaffold-based polysaccharide hydrogel is that it has weak mechanical property. Some inorganic nanocomponents could be used and incorporated to improve the mechanical property, such as inorganic hydroxyapatite nanoparticles; however, this affects the injectability of the hydrogel. Before being implanted, preformed polysaccharide hydrogel scaffolds have a predesigned size and morphology. Several approaches are used to create a preformed polysaccharide hydrogel scaffold, including the lyophilization process, casting process, and 3D printing process. The most promising method for promoting and guiding tissue regeneration is 3D printing.

## 2. Background of Use of Polysaccharide Hydrogel for Bone Defect Treatment

A bone defect is a bone cell lack where it should normally be present. Defects of the bone are caused by infection (osteomyelitis), tumor, or trauma [21,22]. The orthopedic surgeon has tremendous difficulty when it comes to surgical restoration of bone deficiencies [23,24]. In clinical practice, bone abnormalities in the extremities are widespread due to severe trauma, infection, and tumor excision. Bone abnormalities larger than two centimeters cannot mend on their own, necessitating reconstructive surgery. Bone tissue engineering (BTE) plays an important role in critical-sized bone defects. Bone tissue engineering (BTE) requires combinations of scaffolds with several bioactive agents, such as cells, medicine, protein, and other bioactive molecules [25]. Bone scaffold materials should exhibit biomimetic properties [26]. Recently, the scaffold-based polysaccharide hydrogel (SPH) is effectively used to repair defects of the bone [27]. A standard scaffold-based polysaccharide hydrogel includes a two-stage set of bone regeneration in seeding cell and bioactive molecules [28,29]. The first stage involves the insertion of a stem bone cell (e.g., mesenchymal stem cells (MSCs)) with the incorporation of active agents, such as anti-inflammatory medicine and enzymes [30,31]. A scaffold-based polysaccharide hydrogel in a foreign body reaction is known as a soft tissue similar to a native natural extracellular matrix (ECM) [32,33]. The mechanisms of a scaffold-based polysaccharide hydrogel mainly include the following: (1) the polysaccharide hydrogel is low an immunological reaction that has multiple functional groups in their backbone acting as an attachment to prevent the loosening of a bone cell and medicine due to blood flow and give time to the promotion of the bone cell formed into the fracture site [34]. (2) The scaffold-based polysaccharide hydrogel contains mesenchymal stem cells (MSCs) and different protein compounds that express bone morphogenetic protein-2 (BMP-2), transforming growth factor-β (TGF-β), and vascular endothelial growth factor (VEGF) [35,36,37]. (3) The surface of the polysaccharide hydrogel is richly microporous to increase the blood supply. Therefore, the scaffold-based polysaccharide hydrogel mainly has a porous structure to promote intrinsic osteogenic activity [38]. 

## 3. Significance of Scaffold-Based Polysaccharide Hydrogel 

In order to promote better bone regeneration, polysaccharide-hydrogel-based cell delivery and drug delivery have emerged as potential solutions in bone tissue engineering (BTE) and regenerative bone [26,34,38]. Because of its numerous therapeutic uses, bone regeneration has sparked a lot of interest in tissue engineering and regenerative medicine. Although the natural bone has good mechanical qualities, it has low biocompatibility. To encourage improved bone regeneration, bone cell transport using polysaccharide hydrogels has emerged as a promising option for bone tissue engineering and regenerative medicine. They can provide a natural hydrophilic three-dimensional environment conducive to bone cell survival and support new bone growth. This review article focuses on research of polysaccharide-based hydrogel as a carrier for drug, cell, and bone tissue engineering. It introduces polysaccharide-based hydrogel as a scaffold, which provides the bone cell survival environment and is conducive to bone regeneration. The roles of polysaccharide-hydrogel-based bone cell delivery systems in bone repair are discussed briefly in order to better understand the effect of the polysaccharide hydrogel on bone tissue engineering (BTE).

## 4. Architecture of the Natural Bone

Understanding the physicochemical architecture of the natural bone and its pertinent biomechanical characteristics is essential for selecting the best biomaterial. From a nanoscale viewpoint, a bone is mostly made up of collagen strands that are infiltrated and surrounded by minerals at the nanoscale. Four critical components of the bone should be examined and recapitulated as precisely as possible in the logical design of a regenerative nanocomposite for bone cell regeneration [39,40]: (1) composition structure, (2) physical influences, (3) architecture, and (4) biochemical triggers. A multitude of nanostructured materials have been created during the last decade to induce the regeneration of the bone by imitating these four essential properties of the bone. It has been proven that technologies that replicate more than one of these four essential aspects produce better results [41,42]. Material design for bone regeneration applications must consider the architecture of the native bone [41,43]. These materials must promote cellular recruitment, adherence, proliferating, and pro-osteogenic differentiation by providing an appropriate environment. Several methods allow for the precise control of the porosity, topography, and mechanical characteristics of diverse polysaccharide hydrogel scaffold materials [44,45], all of which have shown to be beneficial in the regeneration of bone cells [46]. Providing an appropriate environment for osteogenesis regeneration of the bone is an only important factor to be considered [47]. These polysaccharide hydrogel scaffold materials must be robust, biocompatible, and able to merge with surrounding bone tissues, among many other features, for them to be useful in therapeutic settings [48].

The structure of the bone has a hierarchical organization that spans from the nanoscale to the whole bone level, which is made up of an organic compound (primarily collagen) and an inorganic compound (mostly nanohydroxyapatite) mostly with a hierarchical plate structure [49,50]. The hydroxyapatite (HA) plates are 2 × 25 × 50 (Z, X, Y) nm in size, with 2 nm of carbonate apatite and 3–10 nm of collagen molecules. In the bone, the chemical structure is similar to type I collagen and β-tricalcium phosphate (β-TCP) [51]. Noncollagenous organic proteins (OPN, BSP, and osteonectin) govern mineral deposition by acting as chelation-regulated calcium and phosphorous ion reservoirs and determining the size and orientation of mineral crystals [52]. Because conventional treatments have severe limitations, nanomaterials provide a novel method for bone repair. Nanostructured scaffolds provide cells with a more natural-looking structural support and govern bone cell proliferation, bone cell differentiation, and bone cell migration, resulting in the creation of functional tissues [53]. This review aims to design and categorize nanostructured materials and nanocarrier materials for bone regeneration. Simply replicating the aligned fibers observed in the original collagenous bone architecture is a more direct approach to biomimicry. Plant polysaccharides are macromolecules that are made up of several monosaccharides containing- or β-glycosidic linkages, some of which may be same or different. Starch, cellulose, pectin, and other substances like these are found in plants. Plant polysaccharides are widely distributed, and as a result, the molecular weight and content of polysaccharides from various species vary. Plant polysaccharides have received a lot of interest in recent years due to their substantial bioactivities and suitability for use in medicine and various biomedical applications. Innovative techniques employing aligned nanofibers produced by electrospinning have made it feasible to achieve this level of accuracy in biomimicry [54,55]. Nanofibrous-scaffold-based polysaccharide hydrogel materials provide more potential to control cellular function and drive cell development by mimicking the morphological structure and chemical composition of the natural extracellular matrix (ECM) at the nanometer size [56]. Collagens, fibronectin, elastin, proteoglycans, glycosaminoglycans, laminins, and other glycoproteins make up ECM. Furthermore, polysaccharide hydrogel materials have porosity that improve osteoconductivity and also provide acceptable biocompatibility, biodegradability, and good mechanical strength, which could be used as BTE scaffolds.

The BTE scaffolds must have the following functions: (1) have temporary mechanical strength to support the injured region and fill the vacuum left by deficiencies of the bone, (2) boost circulating precursor cell adhesion and development and enable ECM accumulation on the scaffold surface (osteoconduction), (3) stimulate the ingrowth of the vasculature and bone cell into the porous scaffold; (4) increase osteogenic differentiation and the creation of a new bone tissue through molecular signaling (osteoinduction), (5) promote the incorporation of a native tissue by increasing cell activity (osteointegration); (6) and provide medicines or bioactive substances to help the healing process move along more quickly. Polysaccharide-based hydrogels may be prepared as a porous sponge scaffolds structure, fibrous scaffold structure, and membrane scaffold structure, which are examples of scaffolds that may be treated using traditional or sophisticated methods to produce scaffolds with various topologies.

## 5. Bone Tissue Engineering Manufacture 

The polysaccharide hydrogel scaffolds may be processed in various ways to create porous 3D scaffolds for BTE. The casting process, particle leaching process, gas foaming process, emulsion process, freeze-drying process, electrospinning process, and thermally induced phase separation process are some of the most common processes [57,58]. The “solvent casting and particle leaching” approach entails mixing a solution of biodegradable polymers with water-dissolved ions (e.g., sodium citrate and sodium chloride), which is then casted into a desired bone shape. In the gas foaming process, a gas (typically CO_2_) is delivered to solid polymer discs under high pressure until saturation is achieved [59]. The rapid release of the gas eventually forms a spongy structure in the polymer. In the freeze-drying process, a polysaccharide hydrogel solution consisting of both organic aqueous phases is homogenized and rapidly cooled to maintain the liquid state structure [60,61]. The freeze-drying process removes the solvent and water from the polysaccharide hydrogel, leaving framework scaffolds with a high porosity degree (more than 90%) [62]. The electrospinning process is a method for squeezing a viscoelastic solution into a jet by applying strong electric pressures to overcome internal interaction forces: Nano-/microsized fibers are produced as the solvent evaporates. In the sol–gel method, inorganic material solutions or dissolved metal–organic materials are in suspension forms [63,64].

The phase separation method that originally used to make porous membranes and 3D scaffolds involves a first processing step in which the polysaccharide hydrogel is dissolved or suspended at a high temperature [65,66]. Lowering the temperature causes the solid–liquid phase separation to take place. Finally, sublimation is used to remove the solidified solvent-rich phase from the polysaccharide hydrogel, leaving empty areas that determine the matrix porosity network scaffolds. Other common manufacturing methods include fiber bonding, fiber mesh, powder compaction methods, and melt molding. 

## 6. Nano-Inspired Scaffold-Based Polysaccharide Hydrogel for Bone Tissue Engineering 

Inorganic nanotechnology and polysaccharide hydrogel now offer a tool for designing scaffold-based polysaccharide hydrogel biomaterials with tunable characterizations useful for bone tissue engineering (BTE) and periodontal regeneration [53,67,68]. BTE is a unique strategy for promoting bone defect repair by regenerating the new bone cells that use scaffolds seeded with cells or growth factors of an integrating bioactive cell. It is thought to circumvent the aforementioned difficulties and give an innovative platform in tissue regeneration [69,70]. The polysaccharide hydrogel scaffold comes from natural origin (Table 1) is employed in bone tissue engineering designed to provide structural support; provide an environment conducive to cell adhesion, migrations, growth, and differentiation; and mimic the bioactivity of bone defects [71].

**Figure 2:**
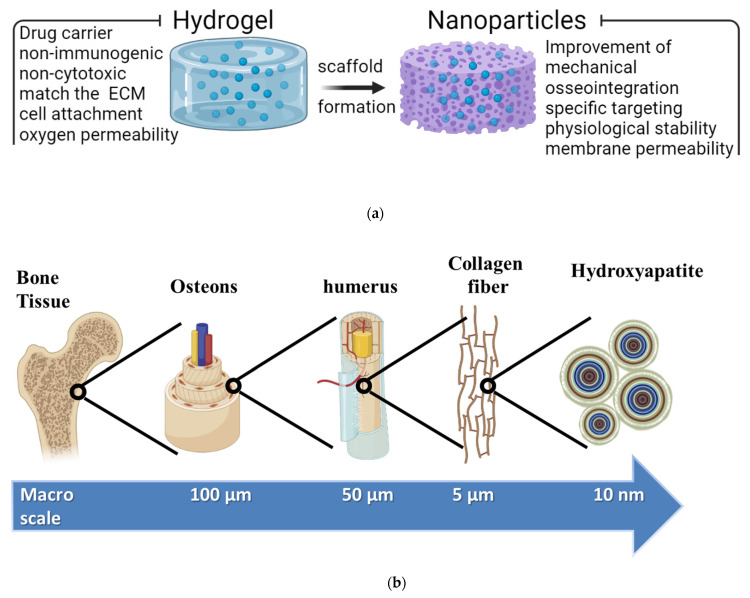
(**a**) Superstructures of a nanoparticle–polysaccharide hydrogel for a tissue engineering scaffold. Nanoparticle–polysaccharide hydrogel systems combine the distinct benefits of their constituent components, allowing them to excel in drug delivery, immunological regulation, detoxification, and tissue engineering, among other uses. (**b**) The hierarchical structure of a bone ranging from microscale skeleton to nanoscale collagen and hydroxyapatite.

Creating a composite scaffold that combines their osteogenic development and three-dimensional matrix hydrogels is important [72]. In [73], conductive nanofibrous-scaffold-based polylactide (PLA) was fabricated with well-distributed NPs of polyaniline (PAn) for bone regeneration. The obtained results confirmed that a different content of PAn NPs in the scaffold stimulates osteogenic bone-marrow-derived mesenchymal stem cells for bone tissue engineering. In [74], a three-dimensional porous humanlike collagen-based nanohydroxyapatite (n-HA) formed inside cross-linked 1,2,7,8-diepoxyoctane (DEO) was successfully fabricated for bone tissue regeneration. Natural bone stroma mainly comprises a (collagen) component and hydroxyapatite component. Because its chemical structure and crystalline characteristics are very close to those of natural bone apatite, hydroxyapatite possesses outstanding biocompatibility, great plasticity, and extraordinary mechanical qualities. Several research studies have used a collagen composite with hydroxyapatite in a scaffold-based hydrogel for bone regeneration. Particularly for bone cell regeneration, hydrogels are of interest for use as composite scaffolds due to their unique configurations and their extensive biocompatibility. Hydrogel materials can be prepared from natural origin materials that are very closes to the natural ECM (Figure 2 and Table 2), such as proteins (fibroin, fibrin, gelatin, and collagen) and polysaccharide compounds (hyaluronan, chitosan, and alginate). In [75], a tripolymer block hydrogel was successfully prepared, which was composed of poly ((ethylene glycol)-(ε-caprolactone)-(ethylene glycol))-based collagen and nanohydroxyapatite (n-HA). According to in vivo results, the biodegradable tripolymer block hydrogel/collagen/nanohydroxyapatite composite showed greater biocompatibility and directed bone regeneration performance in rabbits than those of the self-healing process [76]. In [77], a scaffold-based collagen hydrogel and an alginate hydrogel were used as the main carrier of coculture cells. They demonstrated an experiment series of coculture parameters for human mesenchymal stem cells (hMSCs) and human umbilical vein endothelial cells (HUVECs), which showed that bone cells are pushed toward angiogenesis and osteogenesis depending on the kind of scaffolds. The results showed that collagen provides angiogenesis and osteogenesis, while alginate is useful for osteogenic purposes. A core–shell capsule made of alginate improves the osteogenic capacity of human osteoblast-like MG-63 cells [78]. Since it is biodegradable, biocompatible, and easy to manufacture into an injectable microbead form, sodium alginate is a suitable polysaccharide for the encapsulation and immobilization of a variety of cells in bone tissue engineering [79]. A sodium-alginate-based hydrogel can be employed to transport MSCs and thereby attract endogenous cells via paracrine signaling, and more osteogenic stimuli, such as calcium hydroxyl apatite, are required to restore critical-sized segmental femoral lesions [80]. The calcium, zinc, and strontium ions can be utilized to adjust the characteristics of alginates for the production of composite scaffolds for the regeneration of the bone tissue, because calcium, zinc, and strontium are ions of interest due to their osteogenic qualities [80].

Ideally, in Table 2 is show the origin of polysaccharide came from plants, animals and marine organisms. Optimized polysaccharide hydrogel formulations with nanoparticle for biomedical uses is summarized in Table 3. For example, hydroxyapatite composed of Ca, P, Zn, and Mg for the regeneration of the bone as scaffold (Table 4) need to coincide with the following conditions: (1) being nonimmunogenic and noncytotoxic to avoid inflammatory response in the human body [81]; (2) being osteogenic, osteoconductive, osteocompatible, and osteoinductive for the improvement the bone cell regeneration [82]; (3) strongly matching the natural ECM for easy bone cell attachment, cell proliferation, and eventually osteogenic differentiation at the bone implant site in mimic cells [83]; (4) being able to undergo enzymatic or biodegradation, synchronizing with new bone cell regeneration to allow for new bone growth [84]; (5) possessing architectural stability and good mechanical properties to avoid denaturation during sterilization and load-bearing faults [85]; (6) having an appropriate porous structure and linked porosity, which can be optimized by changing the concentration and wide range of polysaccharide hydrogels and their blends to improve bone cell engagement, regulate the release of entrapped bioactive materials, and enable the transport of nutrients and oxygen within the polysaccharide hydrogels [86]; and (7) providing comfort and simplicity for the patient undergoing injection [87]. As a rigid organ in the body, the bone is able to support and protect various organs but is also able to facilitate mobility [79]. These properties are mainly attributed to the remarkable hierarchical architecture, which is constituted by the soft collagen protein and stiffer apatite mineral, as shown in Figure 2b.

**Table 1:** Natural polymer classification according to chemical structure [88,89].

Polysaccharides	Proteins
Alginate	Collagen
Starch	Gelatin
Cellulose	Silk
Chitosan	Soybean (*Glycine max*)
Hyaluronic acid	Fibrin
Xyloglucan	Albumin
Chondroitin sulfate	Casein
Cyclodextrin	Zein
Dextran	Gliadin
Heparin	Legumin
Kappa-carrageenan	Elastin
Gum polysaccharides	
Pectin	
Pullulan	

**Table 2:** Classification of polysaccharides according to their origin [90].

Plants	Mucilage, Pectin, Hemicellulose, Gums Cellulose, Glucomannan, Starch
Algae	Carrageenans, alginates, galactans, agar
Animals	Cellulose, glycosaminoglycans, hyaluronic acid, chitosan, chitin
Bacteria	Cellulose, xanthan, polygalactosamine, gellan, levan, dextran
Fungal	Yeast glucans, chitosan, chitin, pollulan, elsinan

**Table 3:** Advantages of a nanoparticle–polysaccharide hydrogel for biomedical uses [90].

Application	Advantages of Nanoparticle–Hydrogel Superstructures
Drug delivery	Enhanced protection and stability of the drug Prolonged drug retention and drug release sustained Responsive drug release by internal and external stimuli responsive like pH
Detoxification	Detoxification agent confinement to the site of diseases Retention of prolonged and release of sustained released
Immune modulation	Off-target effects reduction Controlled therapeutic and drug dosages Responsive release of cargo by internal and external stimuli
Tissue engineering	Tunable mechanical properties Localized and controlled delivery of drugs Enhanced bioavailability

**Table 4:** Experiment design of natural polysaccharides loaded with different bioactive materials as scaffolds.

Natural Polysaccharide	Delivery System	Experiment Design	Outcome	Ref
Carrageenan	Nano-HA/gum arabic/k-carrageenan composite scaffold	Analysis of the mineralization process and the expression of osteogenic gene markers by osteoblast-like cells using Western blots	Osteoblast-like cells show significant osteogenic markers without cytotoxicity	[91] 2020
Carrageenan	Ag/carrageenan/gelatin nanocomposite	In vitro examination of antibacterial against human pathogens, i.e., *S. pyogenes* 1210, *S. agalactiae* 1661, and *E. coli*	The antibacterial, drug delivery, and anticancer properties of the novel Ag/carrageenan/gelatin hydrogel	[92] 2021
N-carboxyethyl chitosan/hyaluronic acid-aldehyde	N-carboxyethyl chitosan/hyaluronic acid-aldehyde loaded with nanohydroxyapatite	In vitro analysis for osteogenic differentiation. In vivo analysis for alveolar bone regeneration following dental extractions in rats	Maintaining dimensional alveolar ridge and promoting soft-tissue healing	[93] 2020
Regenerated cellulose (rCL) nanofibers/chitosan (CS)	Regenerated cellulose (rCL) nanofibers/chitosan (CS) hydrogel	Alkaline phosphatase (ALP) and alizarin red (ARS) staining were used to assess osteogenic activity in vivo.	The rCL/CS scaffold promoted biomineralization and improved the viability, adhesion, and proliferation of preosteoblast cells (MC3T3-E1)	[94] 2021
Chitosan/hyaluronic acid	Chitosan/hyaluronic acid nanopearl composite	In vivo Cell Counting Kit-8 and ALP activity assessment for preosteoblastic cells	Upregulation of RUNX2, OCN, and OPN genes. Best results were obtained with 10 wt% and 25 wt% nanopearl	[95] 2020
Chitosan	Chitosan nanohydrogel/poly-ε-caprolactone (PCL) loaded with nanotriclosan and flurbiprofen	In vivo study of the NG on experimental periodontitis (EP) rats	Dual antibacterial and anti-inflammatory effects, which revealed an excellent therapeutic outcome	[96] 2019
Gelatin/alginate	Gelatin–alginate–graphene oxide nanocomposite scaffold	In vivo mechanical evaluation and cell differentiation of MG-63 cells in vitro/evaluation of in vivo cone beam	Enhancement in the expression of osteoblast transcription factors and ALP	[97] 2019
Carrageenan	Carrageenan/whitlockite nanocomposite hydrogel	In vivo evaluation of osteogenic activity in adipose-derived stem cells; immunocytochemical staining	Enhancement of osteogenic differentiation and ALP activity	[98] 2019
Carrageenan	Carrageenan/nanohydroxyapatitecomposite scaffold	In vivo evaluation of osteoblast viability and adhesion by MTS viability testing	Promotion of osteoblast activity without any pharmaceutical medicaments	[99] 2018
Chitosan	Chitosan gold nanoparticles combined with peroxisome proliferator-activated receptor g	In vivo testing of gene transfer on the improvement of osseointegration in dental implants in diabetic rats	Improving the prognosis of dental implants in diabetes patients (bone development and mineralization)	[100] 2017
Alginate/chitosan	Alginate/chitosan loaded with nanohydroxylapatite	QuantiChrom ALP kit and alizarin red staining were used to assess MCT3 cell growth and mineralization in vivo.	Stimulation of MC3T3 cell differentiation and mineralization, particularly at increasing hydroxyapatite concentrations	[101] 2015

## 7. Bioarchitecture–Microribbon Hydrogel

Several techniques are used to manufacture a microribbon hydrogel, such as spinning technology. Spinning hydrogel fibers are formed using poly(ethylene glycol) (PEG) and gelatin microribbons μRBs [102]. Subsequent cross-linking of PEG [103] and gelatin yielded the cross-linked microribbons μRBs to form a scaffold with complex geometries at both the macroscale and microscale [104]. Furthermore, these hydrogels enhanced the survivability, proliferation, and dissemination of adipose-derived bone cells under a variety of circumstances, with the potential to mimic the laminar matrix architecture of periodontal tissue regeneration [105,106].

## 8. Gelatin-Based Microribbons

**Figure 3:**
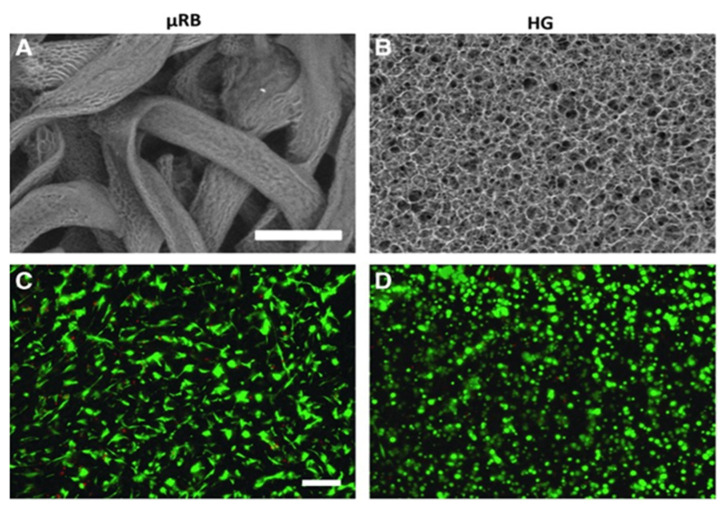
The macroporosity structure of (**A**) traditional HGs and (**B**) gelatin μRB scaffolds had better MSC-based regeneration of cartilage in (**C**) rather than (**D**) Scale bar 100 μm [107], copyright 2019.

Conrad et al. [107] synthesized the macroporous gelatin-based microribbon μRBs by dissolving 12 g of gelatin in 48 mL of dimethyl sulfoxide solvent by stirring at 60 rpm for 18 h at 60 °C. The obtained viscous solution of gelatin was transferred into a syringe and ejected in 3.5 L of ethanol solvent at a speed of 5 mL/h with stirring at 500 rpm. The precipitated microfiber of gelatin was added into acetone solvent and back to ethanol solvent; after being dried, the μRBs of gelatin were formed. Conrad et al. [107] evaluated the potential of a macroporous structure of gelatin-based microribbon (μRB) hydrogels as an innovative 3D matrix for speeding chondrogenesis and fresh cartilage production in 3D with superior mechanical characteristics by hMSCs. Unlike traditional (methacrylated gelatin) HG hydrogels, the μRB hydrogels are intrinsically a macroporous structure and provide cartilage-like mechanical properties. In comparison with natural cartilage, the MSC-seeded μRB scaffold hydrogel had a 20-fold increase in the modulus of compressive and HG scaffolds, and μRBs had a 6-fold increase after 21 days of culture. Compared with HG scaffolds, the μRB scaffolds have increased macroporosity and promote homogenous cell encapsulation with improved viability of the cell, as seen in Figure 3. The macroporosity of the cross-linked μRB scaffolds was significantly interconnected (Figure 3A) compared with that of conventional HGs with smaller porosity (Figure 3B). Furthermore, the μRB scaffolds can support a uniform encapsulation of cells in 3D, and cell viability was evaluated by LIVE/DEAD staining. The cells in μRB scaffolds were extremely viable and distributed uniformly in 3D after 24 h of encapsulation (Figure 3C). In Figure 3D HG scaffolds displayed a good viability of cells but had a round shape due to the physical restrictions of the HG network hydrogel and the absence of a macroporosity network structure. These results support the gelatin-based hydrogel scaffold (μRBs) as good scaffolds for increasing and speeding MSC-based regeneration of cartilage.

Tang et al. [108] used injectable macroporous gelatin-based microribbon μRB hydrogels to support the delivery of the adipose-derived stem cells ASCs and in vivo regeneration of the bone cell using a model of an immunocompetent mouse cranial defect. Bioluminescent imaging was used to determine ASC survival, while micro-CT was used to examine bone repair. A histological investigation was used to measure the rate of degradation and biocompatibility. According to histology and fluorescence imaging, the majority of RBs had deteriorated by the end of 3 weeks. Injectable μRB scaffolds promoted ASC proliferation and bone regeneration at the same rate as implanted prefabricated μRB scaffold controls.

## 9. Poly(ethylene glycol)-Based Microribbons

PEG hydrogels are promising as μRB scaffolds for delivering growth factors to enable cartilage healing. PEG-based microribbons with tunable biochemical, mechanical, and topographical characteristics were developed to construct a 3D cell niche [109]. Ferretti et al. [110] developed a PEG-cross-linked hydrogel with varying genipin concentrations (8, 17.6, and 35.2 mM) of genipin. Cylindrical PEG-genipin cross-linked polymers were implanted into osteochondral defects of the upper extremity muscle of 24 male Sprague-Dawley rats (48 knees). After 5 weeks, the rats were sacrificed; gross, cross-sectional, and histologic examinations were conducted. The in vivo degradation properties of the PEG-genipin cross-linked hydrogel were altered when the concentration of genipin was varied (*p* < 0.01). At a genipin concentration of 8, 17.6, and 35.2 mM, the complete, intermediate, and minimal degradation was observed, respectively. These outcomes indicate that PEG-genipin is biocompatible, and their degradation can be changed in vivo in osteochondral defects according to their concentration.

For the 3D culture cells of patient-derived glioblastoma xenograft (PDTX GBM), Wang et al. [111] developed a biodegradable PEG-cross-linked hydrogel with tunable stiffness values of brain-mimicking biochemical cues. Their findings indicated that PDTX GBM cell proliferation was improved when hydrogel stiffness was decreased, and hydrogels with a stiffness of 240 Pa and below supported the spread cells of robust PDTX GBM in a 3D scaffold. Compared with 2D control, PDTX GBM cells encapsulated in hydrogels showed high drug resistance. On the other hand, drug resistance was improved when hydrogel stiffness was increased. 

Elisseeff et al. [112] synthesized poly(ethylene oxide) and poly(ethylene oxide)-dimethacrylate loaded with bovine articular chondrocytes. Athymic mice were implanted with the loaded hydrogel sample. In total, 12 implants were collected from four mice at 2, 4, and 7 weeks. The chondrocytes survived implantation and photopolymerization, resulting in the formation of neocartilage with 1.5–2.9% and 4–7% weight of collagen and glycosaminoglycan, respectively. Histological analysis revealed the tissue structure resembling neocartilage; on the other hand, safranin O staining showed that glycosaminoglycan was dispersed throughout the hydrogels.

DeKosky et al. [113] proposed a novel approach to encapsulate cells in the cross-linked hydrogels with superior mechanical properties of two combined biocompatible PEG-diacrylate hydrogel-based high-molecular-weight polysaccharides (e.g., agarose). Unconfined compression of hydrogel samples showed that shear modulus had a 4-fold increase in a high-molecular-weight polysaccharide with the PEG-diacrylate hydrogel compared with that in the pure PEG-diacrylate hydrogel (39.9 vs. 9.9 kPa) and almost a 5-fold increase compared with a pure a high-molecular-weight polysaccharide agarose hydrogel (8.2 kPa). The compressive failure strains for PEG, hydrogel-based high-molecular-weight polysaccharide agarose, and pure high-molecular-weight polysaccharide agarose hydrogels were 71% ± 17%, 74% ± 17%, and 15%, respectively. This indicates that, in a high-molecular-weight polysaccharide of agarose with PEG-diacrylate hydrogel-encapsulated chondrocytes, the mechanical strength and properties were enhanced. Moreover, LIVE/DEAD viability cell assays showed the survival of cells in a high-molecular-weight polysaccharide of agarose with a PEG-diacrylate hydrogel encapsulation process. Most agarose/PEG-diacrylate hydrogel-encapsulated chondrocytes stayed viable after 1 week of encapsulation. Furthermore, compared with agarose-encapsulated chondrocytes, glycosaminoglycan synthesis was observed in chondrocytes after 3 weeks of encapsulation. Introducing a new approach to encapsulate cells with a PEG-diacrylate hydrogel-based polysaccharide of agarose with improved mechanical properties is promising for cartilage defect repair. 

## 10. Bioarchitecture Microporous Polysaccharide Hydrogel

Other techniques aimed at mimicking the intricacy and signaling features of bone ECM include the fabrication of microporous polysaccharide hydrogel materials (pores smaller than 2 nm in diameter) [114]. Compared with other biomaterial compounds, a microporous polysaccharide hydrogel has similar microporous network structures to the extracellular matrix (ECM) and has good biocompatibility; thus, it can be used as carrier materials for cells or bone growth to promote growth factors in bone tissue engineering. In addition, its soft texture can reduce the inflammatory response of surrounding tissues and surrounding cells, similar to many biological soft tissues [115]. Thus, microporous polysaccharide hydrogels are suitable candidates to apply in bone tissue engineering BTE application for the treatment of disease and drug-targeted delivery. Bone regeneration involves the processes of chronological integration of cells with the surface of biomaterials, including (1) the protein adsorption from biological tissues and blood plasma onto the surface of a microporous polysaccharide hydrogel scaffold; (2) the signaling of bone cells to the implantation site by cytokines and other growth factors in an attempt to restore normal physiology; (3) the extracellular matrix ECM secretion and bone cell maturation to the lamellar bone after immediate bone implant attachment, thereby strengthening the bone implant bonding [116].

**Figure 4:**
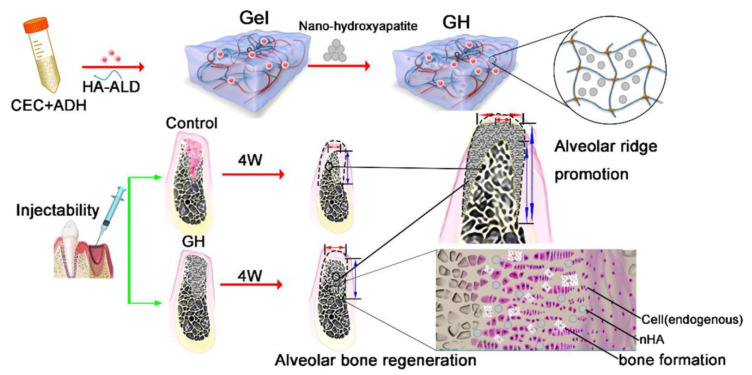
Illustration of the fabrication process of a polysaccharide hydrogel–hydroxyapatite scaffold (GH), which transports the composite to the incisor rat extraction for the investigation of aesthetic alveolar ridge preservation. The alveolar bone deficiency model was created by removing the rat mandibular central incisor, followed by minimally invasive scaffolding into the extraction site. Copyright Elsevier 2020 [121].

Most stem cell transport research has focused on approaches involving cells encapsulated within a nanoporous network using hydrogels (Figure 4). Recent studies have revealed that the restricted nature of this microenvironment has a major impact on cellular function [117]. Microporous polysaccharide hydrogels have been proposed as a solution to this problem. Cells can be implanted in a nanoporous matrix during the annealing of microgels into polysaccharide hydrogels. Cells in polysaccharide hydrogels interact with the nanoporous matrix surfaces but are not encapsulated. Several studies have claimed that the cell spread is superior in microporous-based scaffolds. This method can be implemented to create a wide variety of cell-based polysaccharides by varying the types and concentrations of functional monomers and functional or cell adhesion groups to prepare bone-regeneration-based chitosan hydrogels [118]. Yingqi Chen et al. [119] synthesized the ultraviolet process of functionalized chitosan–methacrylic acid phosphate (CS-MAP) by orderly grafting onto chitosan, phosphopropionic acid, and methacrylic anhydride. The polysaccharide hydrogels have exceptional mechanical qualities and the capacity to regenerate the bone. Bikendra Maharjan et al. [120] added nanofibers of regenerated cellulose into a hydrogel-based polysaccharide of chitosan to enhance proliferation and osteoblast differentiation. A microporous polysaccharide hydrogel has excellent biocompatibility and biodegradation and self-repair advantage and in injectable. The alveolar bone deficiency model was created by removing the rat mandibular central incisor, followed by minimally invasive scaffolding into the extraction site (Figure 4) [121]. After 4 weeks, the osteogenesis and alveolar ridge preservation ability of the polysaccharide hydrogel–hydroxyapatite scaffold was examined by hematoxylin–eosin (H&E) staining and micro-CT. Furthermore, a polysaccharide hydrogel loaded with a hydroxyapatite nanostructure (nHA) is unique in its capacity to stimulate alveolar bone regeneration and the creation of a mineralized matrix in the body without the need for growth factors. One of the greatest hurdles in tissue engineering is maintaining a dimensional bone in attractive regions. Therefore, a polysaccharide hydrogel–hydroxyapatite scaffold (GH) offers a diverse set of bionic scaffolds for maintaining the size of soft and hard structures in the alveolar bone. Overall, our findings suggest a new approach to soft-tissue regeneration and alveolar ridge preservation in clinical implants.

## 11. Microporous Annealed Particle (MAP) Hydrogels

Microporous annealed particle (MAP) hydrogels are a new type of microporous biomaterial created by annealing microgel particles in situ to create a porous bulk scaffold. In vitro and in vivo studies have revealed that microporous annealed particle (MAP) hydrogels promote and increase the proliferative and regenerative activities of bone cells. As a result, combining gene or active pharmaceutics delivery agents with microporous annealed particle (MAP) hydrogels provides a viable technique for bone healing and cell production optimization. This section demonstrated the impact of particle size and stiffness, as well as adhesion potential, on cell surface area and proliferation, and then connected these data with the capacity of cells placed in these scaffolds to get transfected. This section demonstrates the importance of considering microporous annealed particle (MAP) hydrogel properties for proliferation gene transfer and guiding bone cell spreading.

### 11.1. Gelatin-Based MAP Hydrogels

Zoratto et al. [122] utilized gelatin and its cross-linkable derivatives, such as gelatin methacryloyl (GelMA), because of their ECM-mimetic characteristics in biomedical applications. The GelMA microporous hydrogels were inserted into the droplet generator with a continuous phase of oil and surfactant. Moreover, they collected the GelMA microbeads in the oil phase, and the in vitro biological activity of microporous scaffolds of GelMA was investigated by mixing cells of an NIH/3T3 fibroblast. For the control, the live cell number and total cell number were normalized to quantify the biological activity of cell-laden GelMA microporous scaffolds created by photoannealing physically cross-linked microgels. Both beaded GelMA scaffolds increased ~2.8, 4.2, and 7-fold at days 3, 5, and 7 after seeding, respectively. 

Recently, click hydrogel has been used in different biological uses. Many orthogonal approaches have been devised for constructing biomaterial hydrogel samples to promote a “3D” cell culture due to their gentle, cytocompatible, and highly selective reaction kinetics. Owing to the high degree of tunability in orthogonal click chemistry cross-linking reactions, installing a specific biomimicry in an artificial ECM has become possible. In addition to click chemistry reactions, a specific enzymatic reaction (SER) is more frequently utilized to cross-link a network hydrogel and spatiotemporally used to control the hydrogel characteristics. In contrast, a covalent cross-linked adaptable hydrogel by radical polymerization reactions has been employed to construct the viscoelastic component of bone tissue engineering. The fact that covalent adaptable chemistry, enzymatic reactions, and orthogonal click chemistry can all be performed under aqueous and ambient conditions is necessary for sustaining cell viability for in situ encapsulation of bone and steam cells and postgelation modification of polysaccharide hydrogel network characteristics [123].

Isaac et al. [124] used bioorthogonal tetrazine materials in click reactions to in situ form and give click MAP hydrogels based on PEG (i.e., Tz/MAP hydrogels). Clickable PEG-peptide hydrogel MPs containing ECM-mimetic peptides were developed via submerged electrospraying and stoichiometrically regulated thiol–norbornene click chemistry to enable cell attachment and enzymatic breakdown. Unreacted norbornene groups in the MP hydrogel were then employed for bioactive protein functionalization and annealing into Tz/hydrogels through the tetrazine–norbornene click reaction, which is extremely selective and occurs spontaneously without the aid of an initiator or catalyst [125]. The researchers discovered that the clickable particles may be simply applied to a tissuelike defect and annealed into a microporous structure in situ [126].

Furthermore, various hydrogel microspheres may be used to create a click hydrogel of a Tz/MAP hydrogel with heterogeneous properties: tetrazine-modified alkaline phosphatase was coupled to PEG hydrogel MPs combined with nonfunctionalized MPs to make protein-functionalized hydrogel MPs for Tz/MAP hydrogel synthesis. After incubation in calcium glycerophosphate, a biomimetic mineralized/nonmineralized interface was created. Tz/MAP hydrogels were then injected with platelet-derived growth factor-BB (PDGF-BB) and human periodontal ligament stem cells (PDLSCs) during the annealing stage to demonstrate their potential to deliver regenerative therapies, specifically for periodontal tissue regeneration. In vitro analysis revealed good PDGF-BB retention and PDLSC development and distribution. Furthermore, adding PDGF-BB to hydrogels increased PDLSC proliferation by 90% and nearly doubled the average cell volume.

Tz/MAP hydrogel materials appear to be a viable novel delivery platform of stem cells and regenerative factors based on these findings. Caldwell et al. found that MSCs had roughly 95% survivability following a 96 h culture period in vitro using a comparable PEG-based MAP scaffold. Furthermore, the scaffolds permitted cell growth and interaction regardless of the tissue environment and were very adaptable and flexible. Because MAP scaffolds can promote cellular activities in tissue regeneration, the findings suggest that they should be studied further in vitro and in vivo. For example, the shape (spread vs. round) of hMSCs growing in MAP scaffolds made from PEG MPs functionalized with the cell adhesion ligand arginine–glycine–aspartic acid (RGD) was regulated and altered by changing the size of MPs utiliz ing injectable MPs [127]. To accomplish long-term human bone-marrow-derived mesenchymal stem cell (hBMSC) preservation and chondrogenesis, Li et al. [128] produced a tissuelike structure of greater order. The four-arm poly(ethylene glycol)-N-hydroxysuccinimide (NHS) cross-linker forms covalent connections between the microgel building blocks and the surrounding tissue mimic, preserving the vitality and biological activity of the encapsulated hBMSCs. The chondrogenic indicators in gene and glycosaminoglycan (GAG) expression levels were boosted by the microgel construct. Furthermore, positive alcian blue and safranin O staining revealed that the regenerated tissue in the produced microgels had hyaline-like cartilage characteristics. In comparison with both the bulk hydrogel and pellet cultures, immunohistochemistry revealed a favorable distribution and a much higher quantity of type II collagen in the produced microgels. Overall, this tissue adhesive hBMSC-laden microgel construct possesses regenerative medicine and articular cartilage repair potential.

### 11.2. PEG-Based MAP Hydrogels

PEG hydrogels are a promising regenerative medicine platform because they can provide an environment in which donated or endogenous infiltrating cells can rebuild or replace tissues that have been destroyed due to illness or trauma. Furthermore, these systems can be used to deliver therapeutic genes that can guide and/or improve the functioning of transplant or endogenous cells.

At great length, PEG hydrogels were derived from natural and synthetic materials. Therefore, nondegradable PEGs are frequently utilized for encapsulation. Moreover, the tunable viscoelastic features of PEGs resulted in a tissue like permeable membrane with low inflammatory response. 

**Figure 5:**
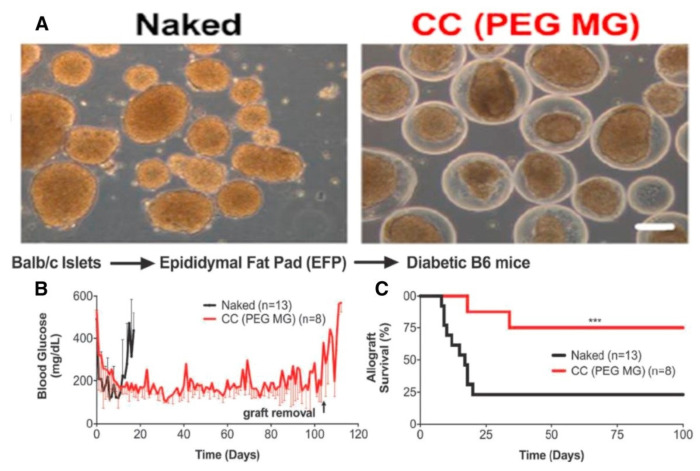
PEG-MAL Matrigel-conformed coated islets transplanted in the epididymal fat pad (EFP) site reverse diabetes in the long term in murine allografts without immunosuppression [129]. Copyright 2018. (**A**) Phase contrast (scale bar, 100 μm) and scanning electron microscopy (SEM); (**B**,**C**) Blood glucose of recipient mice (**B**) and survival (**C**) of 750–1000 IEQ naked (black, n = 13) or CC (PEG MG) (red, n = 8) islets from Balb/c mice transplanted into fully MHC-mismatched chemically induced diabetic B6 mice in the EFP site using fibrin scaffolds without any immunosuppression.

Manzoli et al. [129] incorporated Matrigel into the PEG coating, resulting in poor permselectivity ECM interactions, preventing immune cell penetration and T cell allogeneic priming. PEG and Matrigel were used to create a conformal covering around islets, and a strategy for long-term reversal of diabetes using allogeneic islets implanted in the epididymal fat pad in mice was presented (Figure 5). Furthermore, PEG-based hydrogels are employed due to several advantages, including biocompatibility, structural support [130], and easy functionalization [131]. Several articles have paid attention to artificial ovarian tissue delivery, where it was demonstrated that compared with nonencapsulated follicles, encapsulated immature ovarian follicles in PEG-RGD hydrogels improved the development of primordial follicles and graft survival [132]. Following a subcutaneous transplant of encapsulated ovarian tissue, the estrous cycle was restored in ovariectomized adult mice within 2 weeks. Compared with islets, ovarian follicles are avascular and relatively resistant to hypoxia; therefore, the benefits of immunoisolation methods are maximized.

PEG hydrogels are step-growth hydrogel networks formed using thiolene polymerization [133]. Xin et al. [134] administered photopolymerization and click chemistry to anneal functionalized RGDS and enzymatically degradable PEG microgels into MAP hydrogels using thiolene. During cell-mediated breakdown, the microgel surfaces were reshaped into wrinkles or ridges; however, the scaffold integrity was kept the same. Moreover, compared with nondegradable controls, the proliferation, cell spreading, and secretion of ECM proteins were improved considerably after 8 days of culture, with quicker matrix metalloproteinase degrading (KCGPQGIWGQCK) MAP hydrogels. The relationship between degradability and integrin-mediated signaling was next examined using hMSCs seeded in the MAP hydrogels functionalized with either RGDS or c(RRETAWA), which is specific for α5β1 integrins. More importantly, c(RRETAWA) functionalization increased bone morphogenetic protein-2 secretion overall and per cell; however, this impact was significantly dependent on microgel degradability. Due to the large number of cells in degradable scaffolds, RGDS functionalization resulted in increased total vascular endothelial growth factor secretion. These findings show that integrin-binding peptides may control hMSC behavior in PEG-based MAP hydrogels; however, these results are highly dependent on the microgel building blocks’ sensitivity to cell-mediated matrix remodeling. Future research should be conducted to better improve these materials for stem cell transport and tissue engineering applications.

The water-in-oil emulsion approach was used to create MAP hydrogels that were synthetically tailored to mirror the rigidity modulus of a natural vocalis muscle by Pruett et al. [135]. At day 0, 6 weeks, 4 months, and 6 months, 32 New Zealand white rabbits were administered unilateral MAP injections (n = 16), saline (n = 8), and the clinical standard hyaluronic acid (Restylane-L) injections. Before euthanasia, induced vocal fold vibration was captured with a high-speed camera, and a voice clinician evaluated the characteristic of glottic closure and mucosal wave statistically and subjectively. Furthermore, the scaffold’s durability, immunogenicity, and vascularization were examined histologically. The volume in the MAP gel therapy group remained steady for 6 months after implantation, according to histological analyses. Throughout the implant’s lifespan, immunogenicity was shown to be negligible to nonexistent. Furthermore, immunofluorescence staining within the MAP gel group revealed substantial tissue integration and vascularization histologically. The mucosal wave was unaffected by any of the injected materials, including the MAP gel augmentation. These findings suggest that, when compared with current injectable implants, the MAP gel is a nonresorbable biostimulatory injectable implant that achieves superior tissue integration, stiffness matching, and permanence while maintaining biomechanical function, implying that it could be a promising therapeutic material for glottic incompetence.

Intramyocardial hydrogel injections show promise in noninvasively treating myocardial infarction (MI). Traditional bulk hydrogels, on the other hand, often lack microporous features that allow for fast tissue ingrowth and biochemical signals, which prevents fibrotic remodeling and heart failure. Fang et al. [136], using microfluidic fabrication, created a unique drug-releasing microporous particle (drug MAP) system by encapsulating hydrophobic drug-loaded NPs into microgel building blocks. Then, drug MAP building blocks were created by encapsulating NPs consistently and uniformly and regulating the hydrophilicity and pregel solution viscosity of the NPs. In vitro, forskolin (F) and RepSox (R) have complimentary effects on the functional modulations of cardiomyocytes, fibroblasts, and endothelial cells. The hydrophobic medicines F and R are then loaded into drug MAP in a rat model to form FR/drug MAP for MI therapy. Intramyocardial MAP gel injection improves left ventricular function, which is further improved by FR/drug MAP therapy, promotes angiogenesis, and lowers fibrosis and inflammatory response. This drug MAP platform will be widely used in regenerative medicine and disease therapy as the next-generation microgel particles for MI treatment. Pinnaratip et al. [137] control-clustered silica MPs produced from the aggregation of silica NPs, which were proposed to be included into a catechol-terminated poly(ethylene glycol) bioadhesive (PEG-DA) to create a composite adhesive that may stimulate cellular penetration. The addition of MP to PEG-DA significantly improved the bioadhesive’s mechanical and adhesive properties. The measured values for NP-incorporated and MP-incorporated adhesives showed no significant differences, suggesting that MP and NP were equally successful in enhancing the material qualities of PEG-DA. Most importantly, after being directly exposed to L929 fibroblasts, MP was substantially less cytotoxic than NP. Because of the enhanced porosity within the adhesive network, MP-containing PEG-DA decreased inflammatory responses, raised the amounts of regenerative M2 macrophage to its interface, and improved cellular infiltration when the adhesives were implanted subcutaneously in rats. Control-clustering silica MP improves the performance and biocompatibility of PEG-based adhesives while lowering silica NP cytotoxicity.

In the case of the periodontal regeneration, four distinct cell types compete—namely, periodontal ligament cells, alveolar bone cells, cementoblasts, and epithelial cells. Among these cells, the first three types are responsible for regenerating the periodontal tissue, while the last type of epithelial cells are responsible for soft-tissue regeneration and random bone cell architecture. It is worth mentioning that the higher migration rate of epithelial cells (10 times faster) in comparison with the other periodontal cell types is the reason for observing the formation of the long junctional epithelium in the periodontal therapy. Therefore, the injection of a hydrogel loaded with a specific steam cell is used to limit the infiltration of the epithelial cells [138].

## 12. Polysaccharide and Proteins in BTE

The most widely studied polymers for BTE from natural origin are the peptides glycosaminoglycans (GAGs), hyaluronic acid, collagen/gelatin, chitosan, alginate, silk, elastin, and others. Peptides, like some polysaccharides, include amino groups and carboxylic acid sequence groups that are generally linked to cell adhesion through integrin-binding domains.

Low bioavailability and ion sensitivity are two main limitations that limit the use of peptides. The peptide molecule’s bioavailability is reduced due to limited absorption, metabolic factors like pH, and enzyme-mediated degradation. [139]. Polysaccharide can be used as vehicles for BTE manufacture.

**Figure 6:**
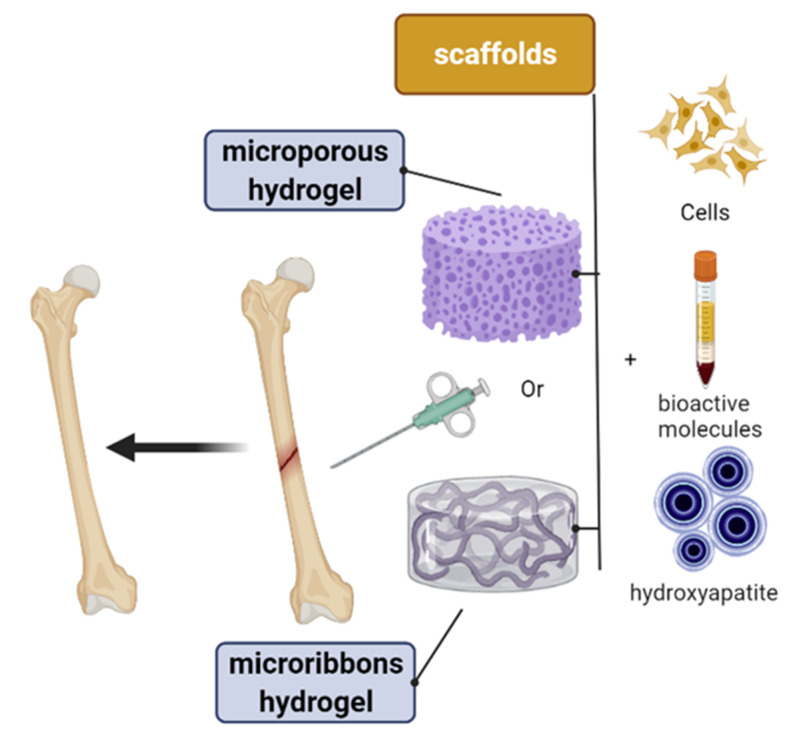
Using natural polymers of polysaccharide as a scaffold hydrogel in bone tissue engineering (BTE).

Besides alginate, chitosan, silk, and hyaluronic acid, collagen and gelatin are the most commonly used natural polymers for BTE. The bioactivity of natural polymers is controlled by the concentration, polymerization conditions, introduction of functional groups that allow for a porosity modulation, and addition of chemicals. Bioactive compounds, such as bioceramics, HA, and other inorganic nanometals, have been incorporated into natural polymers to prepare nanocomposite scaffolds (Figure 6).

**Figure 7:**
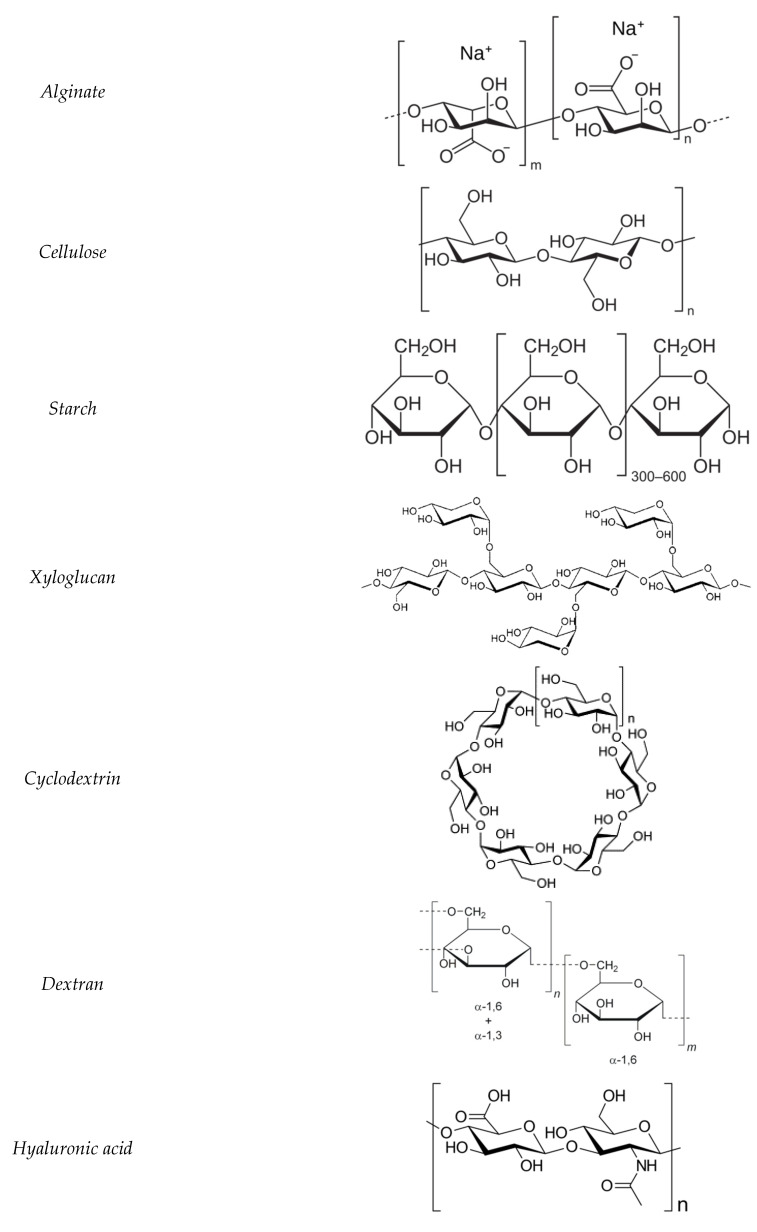

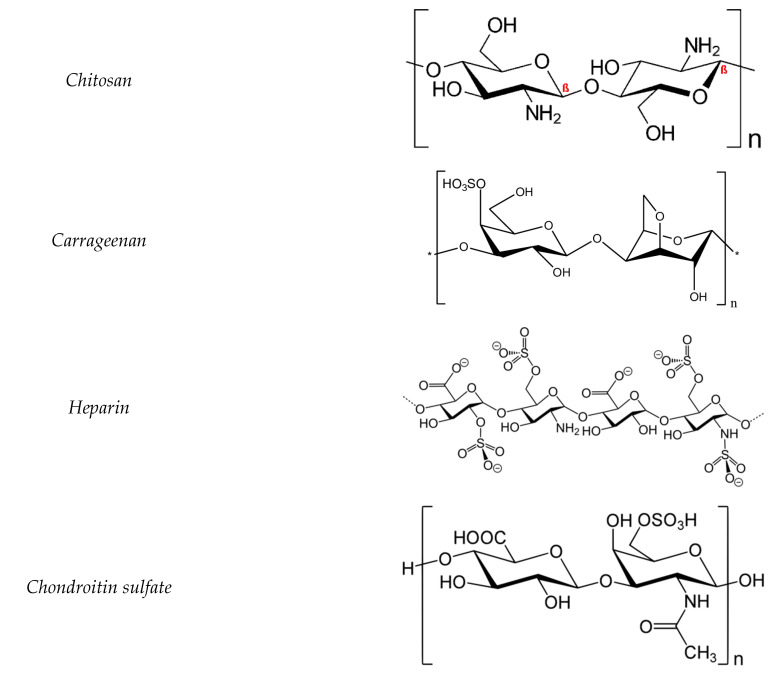
The chemical structural representation of different biodegradable polysaccharide hydrogels for bone-tissue engineering application.

These additions play an important role in improving the scaffold porosity, stability of scaffold structure, osteogenicity, and osteoinductivity [140]. Examples of bioceramics, such as calcium silicate (CaSi) and calcium phosphate (CaP), are incorporated with collagen for bone cell generation. It is known that adding HA to natural polymer scaffolds not only enhances the compression modulus but also offers a bigger and rougher adherence surface, allowing for better bone cell adhesion and proliferation bioactivity. For example, collagen modified with HA with Mg^2+^ substitutions can exert a regulatory effect on the formation process of the bone [141,142], while Zn^2+^ substitution increases the production of genes involved in cell proliferation and osteogenesis [41]. Figure 7 represents the chemical structural representation of different biodegradable polysaccharide hydrogels for bone-tissue engineering application.

### 12.1. Collagen

The collagen protein is made up of a triple helix, which is made up of two identical chains of (α1) and (α_2_) with a slightly different chemical formula. The collagen structure is triple helices, and the physicochemical foundation for their chemical stability has been significantly developed. Collagens are fibrous glycoproteins produced from animals. They are responsible for 28 unique kinds in vertebrates, which are coded by at least 45 various genotypes. Collagen’s amino acid makeup is unusual for proteins, notably in terms of its high hydroxyproline concentration. Collagen is insoluble in rigid fibrous form, which accounts for one-third of all proteins in the human body. The molecules in most collagens are tightly packed together to create long, thin fibrils. These serve as both supporting structures and cell anchors. They provide skin elasticity and strength. In mammals, collagen is the prevalent protein in an animal. Types I, II, III, and IV are the four primary kinds of collagen. The following is a breakdown of the four primary kinds of collagen and its functions in the body. Type I is the most common. This form of collagen, which is made up of densely packed fibers, accounts for 90% of your body’s collagen. Type II exists in elastic cartilage, which cushions your joints and is made up of more loosely packed fibers. Type III is the most common and exists in muscles, organs, and arteries. Type IV is located in the layers of the human skin that are important for filtration. 

Electrospinning can shape collagen into nanofibers [143,144]. Electrospinning collagen for nanofibrous scaffold production has been performed with various solvents, including hexafluoroisopropanol (HFIP), a combination of acetic acid and formic acid [145,146]. Collagen may also be utilized as a hydrogel to create BTE scaffolds. The microstructure of the matrices is influenced by polymerization conditions, such as pH, type of collagen, and their concentration, which impact fibril diameter and density [147]. Collagenous scaffolds’ hydraulic permeability may be modified to optimize not just internal oxygen flow and nutrient exchange but also the construct’s general mechanical properties and cell–scaffold interactions. Under pressure, the capacity of collagen hydrogels to transfer fluids via their interstices varies depending on pore size, number, alignment, distribution, and interconnectivity.

On the other hand, pure collagen scaffold is brittle, and direct culture of a cell produces significant gel shrinkage and geometrical instability [148]. In addition, it suffers from a lack of bioactivity to promote cell bone formation and a lack of mechanical strength to support bone regeneration, necessitating the addition of polymers and other biomolecules to increase osteoinductivity [149]. For example, collagen is poor in terms of Young’s modulus, which can be improved by cross-linking with synthetic polymeric materials [150]. As we know, bioactive glasses (BG) promote both angiogenesis and osteogenesis. Architecturally, BGs are based on the SiO_2_–CaO–P_2_O_5_ alloy [151,152].

The presence of bioactive glasses forms collagen composites with great performance in emulating bone formation. If the release of Ca, P, and Si causes Ca and P to precipitate at the implant’s surface, resulting in the creation of amorphous Ca-P crystals, the crystals become hydroxycarbonate apatite after being dehydrated (HCA) [153]. After soaking in simulated bodily fluids (SBFs), wollastonite (CaSiO3) releases Si and Ca ions, promoting osteogenic differentiation and cell proliferation, as well as causing the deposition of apatite on the bone surface [154,155]. To increase the mechanical characteristics of collagen and the structural stability of osteointegration, collagen may be combined with other materials, such as carbon nanotubes (CNTs), which have been proven to increase collagen composites’ tensile strength, stress resistance, and apatite deposition capability, as well as MSC osteogenic growth [153,154,155]. 

The interaction of preosteoblasts, osteoblasts, and stem cells with collagen inside BTE constructions is a complex biological function of collagen scaffolds that has been identified in vitro and confirmed in vivo. Because of its capacity to expand without dissolving and integrate hydrophobic pharmaceuticals, an injectable collagen hydrogel can efficiently transport bioactive molecules, such as chemicals, proteins, and nucleic acids. It also has a tunable breakdown rate that allows for a controlled release. The release characteristics of bFGF from collagen hydrogels were examined in terms of seeded MSC proliferation and osteogenesis [156]. As a result, the best bFGF dose (10 ng/mL) for making highly stimulatory constructions was discovered. After in vivo ectopic implantation, spatial immobilization of bone morphogenetic protein-4 (BMP4) into collagen–PLGA hybrid platforms increased Ca deposition and expression of osteogenic marker genes, according to another research (such as type 1 collagen, OPN, and OCN) [157]. Furthermore, silicified collagen scaffolds loaded with SDF-1 produced a bone after subcutaneous implantation [158]. The results of the in vitro transwell migration studies revealed that larger concentrations of released factor promoted migration in both MSCs and endothelial progenitor cells (EPCs), and cell-free SDF-1 containing hydrogels encouraged cell homing in vivo and improved blood vessel development.

New possibilities for curing periodontal disease have emerged as a result of recent advances in science, technology, engineering, and mathematics (STEM). Tissue engineering is an example of such advancement, in which cells are combined with scaffolds and bioactive chemicals to try to reconstruct tissues [159]. Periodontal regeneration, as opposed to periodontal repair, aims to restore the periodontal complex’s overall structure and function [160].

Some drawbacks of traditional scaffold materials, such as nonspecific targeting, inadequate physiological stability, and limited cell membrane permeability, may influence the direct delivery of medicinal chemicals. To compensate for the poor pharmacokinetics of such medicines, supraphysiological dosages are usually required, which increase the risk of side effects [161]. Nanotechnology has now made it possible to create structures that are the same size as naturally existing tissues, ushering in a new age for TE/RM [162]. Nanoscaffolds may be made to look and feel much like tissue-specific ECM.

The small size of NPs allows them to respond quickly to environmental stimuli, such as ultrasounds, magnetic fields, pH, and X-ray irradiation. Drugs, genetic material, and biological variables may all be delivered in a regulated manner using nanoscaffold materials, both systemically and locally [162]. Nanoscaffolds can help stabilize bioactive substances by encapsulating or attaching them to surfaces, facilitating molecular internalization, directing their distribution from cells, and controlling biological factor release at the appropriate target [161].

NPs’ ability to produce controlled and sustained results is largely due to their small size and high specific surface area [163]. As a result, they might be used as stimulus–responsive delivery vehicles for chemically or physiologically active compounds, triggering delivery in response to an external signal [161,163,164]. Nanoscaffolds have a high drug loading capacity, high drug loading particle mobility, and good in vivo responsiveness to surrounding tissues [163]. They can be used to mark cells so that they can be tracked and monitored in real time [162,164]. NPs can also help with osseointegration, osteoconduction, and osteoinduction [164]. 

Optimized scaffold formulations for periodontal regeneration should ideally satisfy the following criteria:

(1) Nonimmunogenic and noncytotoxic to prevent an inflammatory reaction;
(2) Improved bone regeneration by being osteoinductive, osteoconductive, osteogenic, and osteocompatible;
(3) To the greatest extent possible, replicating the natural ECM to aid cell adherence, propagation, and eventually osteogenic differentiation at the implant site;
(4) Endogenous enzymes or hydrolysis degradable, synchronizing with new bone ingrowth to provide sufficient space for new bone formation;
(5) For repairing load-bearing defects and avoiding denaturation during sterilization, structural stability and mechanical strength are required;
(6) Suitable pore size and interconnected porosity, which can be improved by varying the concentration and variety of polymers and cross-linkers, to improve cell interaction, control the release of encapsulated bioactive factors, and allow the exchange of nutrients, oxygen, and metabolic waste within the hydrogels.
(7) Patient compliance and injectable capacity to minimize discomfort and simplify the operation [165].

Polysaccharide hydrogels are soft materials with a three-dimensional network structure, water absorption, and chemical and physical characteristics that may be adjusted [166]. For bone and periodontal tissue regeneration, natural-polysaccharide-based synthetic hydrogels with micro-/nanostructures have been demonstrated to mimic the chemical and physical characteristics of natural ECM [167,168]. Hydrogels are characterized as three-dimensional networks with a high water content because of the existence of hydrophilic functional groups that fill the space between macromolecules [169,170]. One of the reasons for their appeal in the biomedical world is their highly hydrated nature, which resembles the ECM [171]. Ester or amide creation, radical polymerization, Schiff base, Michael addition, and disulfide cross-linking are all methods for fabricating these types of biomaterials [172,173]. 

Nowadays, any research project aimed at developing and using ecologically friendly goods made from natural raw materials must have a green and renewable component [174]. Bio-based materials are derived from agricultural commodities and food waste. As a result, bio-based materials are emerging as novel materials in various applications that utilize renewable resources and address environmental concerns [175]. Polysaccharides and proteins are organic macromolecular substances generated by animals, plants, and microbes in nature [176]. 

Polysaccharides also have biological properties, such as antioxidation and anticoagulation, which can help the human immune system respond more effectively [177]. They also have a unique chemical variety and flexibility and complex architectures that are not seen in other polymer types. In the following sections, we will look at how these natural polysaccharides may be used as vehicles for tissue regeneration. Injectable hydrogels have a long history of usage in biomedicine, particularly as intra-articular drug delivery methods [178,179]. They have been employed in the regulated release of growth hormones, chemotherapeutic medicines, and antibiotics to specific cells, among other things [180]. Several biopolymeric polysaccharides, including chitosan, alginic acid, and hyaluronic acid, are useful ingredients for this function [181].

### 12.2. Alginate 

Alginate is used in biomaterial applications in the form of sodium alginate salt and is a natural polysaccharide made up of L-guluronate (G-blocks) and (1,4)-linked-D-mannuronate (M-blocks) produced from brown algae [182]. The mechanical strength and stiffness of the produced hydrogel-based alginate were affected by the G/M ratio, because of its good degradability, biocompatibility, and minimal immunological stimulation [182]. 

Photo-cross-linked-hydrogels based on alginate and loaded with BMP-2 and MSCs were shown to successfully stimulate osteogenic differentiation of stem cells, resulting in greater bone repair with a new bone [101]. Additionally, cell sticky RGD peptides may be converted to hydrogels based alginate to enhance adhesion, proliferation, and spread of cells [183,184]. For aspirin release to a bone defect therapy, a thermosensitive-hydrogel-based alginate and tricalcium phosphate nanocomposite (TSAH/-TCP) were developed, which have a lot of potential bone regeneration [185]. Thermosensitive hydrogels based on alginate, poloxamer, bioglass, and silk fibroin can be used as a potential injectable biomaterials for BTE [186]. An injectable thermosensitive alginate hydrogel (TSAH) loaded with rhBMP-2 could induce ridge augmentation and mineral deposition [187]. The ability of alginate-based hydrogels to preserve the structure of regenerated tissues is generally limited by their mechanical qualities (compressive modulus, 1–8 kPa). To provide a suitable mechanical strength for better support of the regenerated tissues, inorganic fillers were added to alginate hydrogels [188]. Another example, chitosan/alginate/PLGA hybrid scaffolds loaded with IGF-1, BMP-6, was found to generate activated cementoblast proliferation and osteoblastic differentiation [189]. Osteogenic production and AB defect healing, human GMSCs, and bone hBMMSCs can be enhanced by a seeded cell into a silver lactate (SL)-containing RGD-coupled alginate hydrogel scaffold [190]. Recent clinical research using multilayered films made of thiolated alginate and sodium salt of carboxymethyl cellulose polysaccharide for intrapocket metformin administration found that they successfully treated mild periodontitis [191]. For the treatment of periodontal infections, calcium-deficient hydroxyapatite (CDHA) NPs incorporating gelatin–alginate (GA) NPs loaded with tetracycline were shown to give prolonged tetracycline release [192]. In rats, chitosan/alginate hydrogels containing the PTH peptide, PTH(1–34), and nanohydroxyapatite (nHAP) resulted in increased osteogenic differentiation of BMSCs, indicating a novel method for tissue engineering and regeneration [193]. Silver NP-loaded hydroxyethylacryl chitosan (HC) and sodium alginate (SA) showed promise in contemporary wound dressings with antibacterial properties and regulated medication release [194]. Polyvinyl alcohol sodium alginate (PVA-SA) films with hydroxyapatite (Hap) NPs were produced for regulated antibiotic release in the treatment of bony periodontal deformities. HAP NPs promoted periodontal regeneration, whereas amoxicillin promoted the healing of the infection [195]. The osteogenic development of human dental pulp MSCs was enhanced by an alginate–Matrigel hydrogel containing bioactive glass MPs [196]. In vitro, a sodium alginate/hydroxyethylcellulose/hydroxyapatite hydrogel improved hMSC viability and proliferation [197]. In vivo, it stimulated the creation of a new bone to heal the lesion [198]. The cystamine-functionalized sodium alginate–pluronic F127 (ACP) thermoresponsive hydrogel enabled easy encapsulation and controlled release of fibroblasts, making it an appealing biomaterial for cell transport in tissue regeneration [199]. An alginate hydrogel functionalized with synthetic E-cadherin was utilized in a recent work to investigate its role in enhancing PSC attachment, survival, pluripotency maintenance, and differentiation ability [200].

### 12.3. Cellulosic Plant

In general, cellulose is came from several plants, is a major structural component of the primary cell wall of green plants and is the most common polysaccharide compound made up of a linear chain of D-glucose units connected by a b(1/4) linkage. It has no taste and no odor and is insoluble in most organic solvents and water. It is a hydrophilic substance with strong intermolecular hydrogen bonding and van der Waals forces, making its dissolution difficult [175]. Cellulose is a cheap and renewable resource. Biomaterials, such as vegetables, fruits, plants, trees, and biowaste, include the most prevalent natural substance, making cellulose the most abundant natural polysaccharide in nature. Cellulose is one of the safest materials on the planet, encompassing the following benefits: biocompatibility, biodegradability, renewability, high mechanical strength, and environmental friendliness [201]. Cellulose is a fantastic beginning material extract from different plants such as cotton is 99% cellulose, but its applicability is limited due to the difficulty of dissolving it. A chemical reaction into different derivatives is another way to broaden the uses. The hydrogen bond of cellulose is a unique feature of cellulose that allows hydrogels to maintain their structure without the need for cross-linking chemicals [202]. Because of its great biocompatibility, cellulose and its derivatives have been widely employed in biomedical applications [203,204]. For instance, simvastatin was loaded in TiO2 nanotubes, and a thermosensitive chitosan–glycerin–hydroxypropyl methylcellulose hydrogel (CGHH) was then layered on top of these nanotubes in a study conducted by B. Li et al. These constructs showed an enhanced capacity of osteogenesis at a normal body temperature and antibacterial properties in the presence of infection and were considered promising materials for application [205]. 

A thermosensitive hydrogel made of chitosan/hydroxypropyl methylcellulose/glycerol exhibited biodegradability, thermosensitivity, and high fluidity, as well as minimal cytotoxicity and controlled release, indicating that it might be used in biomedical applications [206]. Tissue engineering, wound dressing, and drug delivery might all benefit from the creation of a thermosensitive-hydrogel-based chitosan/carboxymethylcellulose/scleroglucan nanocomposite [207]. Thermosensitive nanohydroxyapatite (nHA) hybrid methylcellulose (MC) hydrogels might be used as a carrier for BMSCs, resulting in osteogenic differentiation and bone repair and a therapeutic approach to bone fractures [208]. At 14 days after implantation, injectable thermosensitive nanofiber-hydrogel-based chitosan/TEMPO-oxidized cellulose demonstrated anti-inflammatory or wound healing (M2) macrophage. Furthermore, adding TOCNF to the CS hydrogel might greatly increase its biocompatibility as a biomaterial for biomedical applications [209]. 

### 12.4. Starch

One of the most significant polysaccharides is starch, which is a natural and renewable polysaccharide that has the following properties: safety, nontoxicity, wide availability, cheap cost, high biocompatibility, biodegradability, and nonimmunogenicity [210]. Generally starch is a plant polysaccharide (Table 1 and Figure 7). Starch is found in intracellular granules and serves as a polysaccharide storage agent rather than as a structure-building component. In plant leaves, photosynthesis occurs in the chloroplasts, where starch is created. It is kept as tiny granules in these regions of the green plant. The plant sections that either need the energy or act as energy storage organs receive absorption starch by hydrolyzing it at night (e.g., grains for cereals and roots for tubers). Starch is stored in these storage organs as water-insoluble granules in amyloplasts. These granules gradually fill up with starch, which is subsequently used as a source of energy during germination. One starch granule or a group of starch granules can be stored within an amyloplast. Given that the size, shape, and architectural details of the starch granules vary depending on their botanical source. Starch does serve as a significant structure-building and -stabilizing carbohydrate in the human diet, in contrast to what is the situation in plants. Focus will be placed on the structure, chemistry, and functionality of starch in biomaterial scaffold applications. Native starch compounds include amylose and amylopectin in 15–30% and 70–85%, respectively, with the proportions changing according to cultivars, growing circumstances, and harvesting procedures. Starch’s characteristics are heavily influenced by the molecular composition and structural organization of its constituents and have a huge impact on the material’s wide range of uses [211,212,213]. Amylose and amylopectin are two polydisperse-D-glucose polysaccharides of high molecular weight that make up starch. Amylose is a mostly linear polysaccharide with many bonds and relatively few branching points. Interglucan interactions, such as entangling and proximal alignment, are possible, thanks to their long linear nature. On the other hand, amylopectin has numerous linkages, leading to a highly branched structure organized in clusters of short branch chains, resulting in a macromolecular organization within starch granules that is rather compact [214]. Starch is a typical hydrophilic macromolecule with hundreds of hydroxyl groups, but due to strong hydrogen bonding between the molecules, its solubility in cold water is very limited. Starch may be given new functions, and its applicability can be broadened by adding new groups to its chains [215]. Chemical changes effectively increase the number of reactive sites that may interact with drug molecules [216], allowing modified starches to be readily absorbed in GIT environments. To acquire modified starches, native starches are often partly hydrolyzed or cross-linked or acetylated or hydroxypropylated or oxidized. Modified starches have been utilized to load various nanocarriers with medicinal medicines against illnesses in recent decades due to their well-designed architectures [217]. A nonionic starch derivative from hydroxyalkyl starch has great application performance due to their high hydrophilicity and high viscosity stability. Hydroxyethyl starch and hydroxypropyl starch have been widely produced and used, while hydroxybutyl starch has received less attention, as shown in a study by Dang et al. [218]. A temperature-sensitive intelligent hydroxybutyl chitosan with a lower critical solution temperature (LCST) of 38 °C created a viscous liquid with high water solubility that could be injected when the temperature dropped below 38 °C. It finished as a solid gel form with good biocompatibility when the temperature was above the LCST [219]. Grafting sensitive hydrogel-based starch or pH-responsive hydrogel-based starch might be a good way to make smart hydrogels that can be used as drug delivery systems de novo [220]. A chitosan gel filled with starch micro-/nanohydrogels served as a dual delivery platform or smart scaffold for tissue engineering [221]. Villanueva et al. developed a method to improve the stability of Cu-NPs. Cu-NPs were produced and subsequently integrated into a starch hydrogel for antibacterial applications after being coated with silica NPs [222]. Compared with a plain hydrogel, the oxidized starch/ZnO nanocomposite hydrogel had more swelling and antibacterial characteristics [223]. Ibuprofen was put into a new oxidized starch/CuO nanocomposite hydrogel for drug delivery applications. The nanocomposite hydrogel demonstrated sustained and regulated drug release in vitro, which improved with a greater CuONP concentration [224]. The new kappa-carrageenan (CA)-coated starch/cellulose nanofiber (CNF) hydrogel has outstanding mechanical characteristics, a variable degradation rate, and the capacity to clot blood, making it a promising option for hemostatic applications [225]. 

### 12.5. Xyloglucan 

Xyloglucan is a natural polysaccharide derived from the of tamarind seed (*Tamarindus indica Linn.*) seeds of a tropical plant that has been grown in the Brazilian Northeast for over a century. This polysaccharide is neutral hemicellulose with a (1-4)-linked D-glucan backbone chain that is partially substituted with D-xylopyranose or 2-O–D-galactopyranosyl–D-xylopyranose at the O-6 position of D-glucopyranosyl residues [226,227]. All land plants have the matrix polysaccharide xyloglucan in their cell walls. A hemicellulose, xyloglucan has side chains with xylose, galactosyl, and fucosyl substituents. Fucosylated xyloglucan, which plays a structural role in plant cell walls, and non-fucosylated xyloglucan, such as that obtained from tamarind seeds, which plays a storage role, have both been detected in plants. In both food and cosmetics, xyloglucan is a frequently used addition that serves as a thickening and stabilizing agent. Different structural-activity connections have been established because side chains are crucial to the polymer’s conformation and, consequently, to how it interacts with other polysaccharides. For instance, thiolation of xyloglucan has been demonstrated to enhance its bioadhesion and drug penetration without altering the final gel characteristics. The use of enzyme-degraded xyloglucan gels as delivery systems for drugs in the nasal and rectal cavities has also been investigated. The polymer’s structural conformation resembles that of mucin, which accounts for its mucoadhesive characteristics. This mucoadhesive characteristic creates a barrier between the mucosal layer and foreign particles. Xyloglucan has been described as an appealing and useful natural polymer in drug delivery tests because of its mucoadhesive and in situ gelling characteristics [228]. Xyloglucan has been used extensively in the pharmaceutical industry, including research into drug delivery by oral, rectal, pulmonary, ocular, and nasal routes. A collection of articles on xyloglucan-based drug delivery systems, including thermosensitive hydrogel and thermoreversible hydrogel in situ for biomedical applications, was recently published [229]. As a result, this polysaccharide has a wide range of applications and should be investigated by academics. However, no xyloglucan-based polymeric nanocapsules have been produced yet. The chemical alteration of xyloglucan can improve its physicochemical characteristics, such as thiolation of xyloglucan, promoting bioadhesiveness, and drug penetration [230]. A xyloglucan/hydroxybutyl chitosan-based composite hydrogel with antibacterial activity against *E. coli*, *P. aeruginosa*, and *S. aureus* showed great promise in in vivo burn wound healing [231]. The ability of xyloglucan hydrogels to create microporous interconnected three-dimensional networks is critical for cell encapsulation in regenerative medicine applications [232,233].

### 12.6. Cyclodextrin

Cyclodextrins (CDs) are harmless cyclic oligosaccharide of the glucose family made up of units of D-glucopyranose interconnected by 1–4 linkages that were discovered in 1903 by Franz Schardinger using a bacterial digest (*Bacillus macerans* and *Bacillus amylobacter*) of starch in potato. CDs are made by cyclodextrin glycosyltransferase, which degrades starch in a very simple enzymatic process (CGTase) [234]. Cyclodextrin has the shape of a truncated cone or doughnut with two open ends because of the chair conformation of glucopyranose units. They have six, seven, eight, or more cyclodextrin-(1,4)-linked-D-glucopyranose units [235,236]. They were used in many industries, including the chemical industry, cosmetics, and food, due to their good biocompatibility and lack of biotoxicity [237]. The outside of a cyclodextrin molecule has a hydrophilic character, whereas the inside has a hydrophobic character. The outer distinctive characteristic is due to the presence of primary hydroxyl groups at the narrow edge and secondary hydroxyl groups at the wider edge, whereas the lipophilic cavity is due to the oxygen atoms and skeletal carbons of the glycosidic connections. [238]. One of the most commonly utilized native cyclodextrins in the production of ICs is β-cyclodextrin (BCD) [239] (water soluble at a temperature of 25 °C = 18.5 mg/mL). As bioadhesive dual-drug nanocarriers, wheat germ agglutinin (WGA)-conjugated liposomes with surface-grafted cyclodextrin (WGA-liposome-CD) were used, which represented new cytoadhesive possibilities for delivering numerous medicines with long-term therapeutic action to oral cells for targeted drug delivery [240]. By embedding phosphorylated cyclodextrin (CD-PH), cyclodextrin (CD), and chitosan into the well-studied chitosan/glycerophosphate system (CS/GP), CD-PH, CD, and chitosan were produced, which showed significant potential applications in dual-drug delivery systems (hydrophobic and hydrophilic) [241]. At low asiaticoside (AS) concentrations, thermally induced in situ gels containing AS in sulfobutylether-CD/chitosan NPs (SBECD/CS NPs) were shown to be nonirritant to HPDLCs. Furthermore, our potential in situ gel formulations containing AS may stimulate the production of type I COL [242]. Antimicrobial photodynamic treatment using encapsulated cyclodextrin NPs and methylene blue irradiation by laser or LED proved successful in reducing multispecies biofilms consisting of early colonizing bacteria [243].

### 12.7. Dextran 

Dextran is a commercially available polysaccharide that is generated by bacterial strains. Dextran has been widely used in medicine and pharmacy as a blood plasma substitute, bioantifouling materials, and other applications due to its biodegradability, biocompatibility, nonimmunogenic, and nonantigenic characteristics [244,245]. Dextran also has a low degree of branching, making it an excellent starting material for creating well-defined derivatives. The dextran/poly(N-isopropylacrylamide) (Dex/PNIPAM) copolymeric matrix loaded with thermoresponsive graphene quantum dots (GQDs) showed no inflammation and significant stromal cell infiltration, demonstrating that the synthesized drug carriers did not harm the nerves or tissues and were only responsible for pain management [246].

### 12.8. Hyaluronic Acid

Hyaluronic acid (HA) or hyaluronan is made up of alternating units of a repeating disaccharide called 1,4-D-glucuronic acid and β-1,3-N-acetyl-D-glucosamine. HA is a nonsulfated glycosaminoglycan that is the primary component of ECM throughout the body [247]. In the vitreous humor, the molecular weight of HA as a hydrated polyanionic macromolecule varies (100–8000 kDa). The biochemical and structural characteristics of HA allow it to regulate a variety of physiologic activities, including wound healing, morphogenesis, ECM structure, and signaling pathways [247,248]. Meyer and Palmer coined the phrase “hyaluronic acid” after successfully extracting the chemical from cow vitreous humor and naming it hyaloid (meaning vitreous) and uronic acid, one of the sugar molecules that make up the polymer [249]. Based on the hyaluronidase enzyme activity, the tissue half-life of HA ranges from an hour to days [250]. Because of these benefits, HA and its derivatives have become a popular medicinal product during the last three decades [251]. Furthermore, recent advancements in the field of cell treatment have identified HA hydrogels as important biomaterials in tissue engineering [252,253]. As a result, HA derivatives are formed, which may be classed as alive or monolithic [254]. In the presence of biological molecules in various cells and tissues, living derivatives may establish de novo covalent connections, whereas monolithic HA has been changed in its terminal portions and can no longer form chemical attachments. Living HA derivatives boost in vivo and in vitro 3D preclinical and clinical trials [255]. There has been a lot of interest in developing a live HA derivative for therapeutic application in recent years. Glycosan Biosystems provided a live HA derivative with these advantages [256]. HA and its derivatives have been proven to have anti-inflammatory effects and can inhibit immune cell activity [257]. The cytocompatible hyaluronic-acid-based gelatin hydrogel with biphasic calcium phosphate (BCP), tricalcium phosphate (TCP), and ceramics might stimulate osteogenesis by upregulating the expressions of the bone-related genes COL1, RUNX2, ALP, and OPN. The rabbit femur defect model indicated that the implanted HG/TCP/BCP plug promotes bone regeneration with a high rate of collagen dispersion and ALP and OPN expressions, suggesting that it might be used in dental applications for one-step socket preservation [258]. Hyaluronic-acid-based scaffolds that may be injected could help in bone repair [259]. Moreover, HA is recognized for its osteoconductive properties, ability to induce angiogenesis, and ability to regulate immunological responses [260]. The hydrogel (xanthan (2%) and HA (1%) containing Arenicola marina’s hemoglobin (M101) exhibited encouraging results and might improve periodontitis therapy using a noninvasive method [261].

### 12.9. Chitosan 

Chitosan is positive-charge semicrystalline polysaccharide composed of 2-amino-2-deoxy-D-glucosamine and 2-acetamido-2-deoxy-D-glucosamine units arranged randomly all across the polymer structure [262]. Deacetylation, also known as alkaline hydrolysis or enzymatic hydrolysis of chitin, can form chitosan. Invertebrates (crustacean shells and insect cuticles) and fungi (yeasts envelopes, cell walls, green algae, and mushroom) both have chitin [263]. The physicochemical and techno-functional characteristics of chitosan are influenced by its molecular weight (Mw), degree of acetylation (DDA), purity, and sequence of the acetamido and amino groups [264]. Low-Mw chitosans (22 to 1800 kDa), for example, are more water soluble and have superior antimicrobial capabilities than high-Mw chitosans [265]. When chitosan is combined with negatively charged polymers or tiny-molecular-weight substances in solution, such as HA, electrostatic complexes are formed [266]. Many forms of chitosan-based hydrogel scaffolds are membranes, films, microfibers, nanofiber tubes, microspheres, and nanospheres [267]. Solvent casting, freeze drying, electrospinning, NP leaching, gas foaming, 3D printing, and other methods can be used to manufacture these biomaterials, or they can also be used in combination [268].

### 12.10. Carrageenan

Carrageenans are extracted from red seaweeds from the family of sulfated polysaccharides with high molecular weight, similar to ECM-derived glycosaminoglycans. Carrageenan macromolecules are made of 1,3- and 1,4-glycosidic linkages that connect alternating units of 3,6-anhydro-galactose and D-galactose [269]. Carrageenan is a thickening, stabilizing, and gelling ingredient and a fat substitute commonly used in cuisine [270]. Furthermore, the anionic polysaccharide’s commercial importance is demonstrated by its application in the pharmaceutical industries, textile, and cosmetic. The use of carrageenan as additive of food is usually considered safe. In numerous studies, carrageenan was found to have extremely low toxicity and no teratogenicity in monkeys, guinea pigs, mice, and rats [271]. However, when formed in nanometric complexes, some investigations have raised concerns regarding its safety. According to Catanzaro et al., carrageenan caused macrophage cytotoxicity but not lymphocyte cell death. When given in large dosages, it creates a lot of lysosomal storage, which leads to lysosome rupture and cell death [272]. Another study found that carrageenan/chitosan NPs at concentrations ranging from 0.1 to 3 mg/mL did not affect L929 fibroblasts produced in vitro [273]. The three-dimensional structure of carrageenan encourages osteoblast development and adhesion [274]. When coupled with nanohydroxyapatite, it boosts osteoblast activity [275]. Adding carrageenan to the scaffold structure improves the compressive strength of a hydroxyapatite–collagen composite hydrogel. [276]. Low cytotoxicity against human osteoblast cells and high antibacterial activity against *Pseudomonas aeruginosa* were found in blended carrageenan hydrogels with different nanohydroxyapatite ratios. Furthermore, exposing cells to carrageenan nanocomposite hydrogel and whitlockite NPs has been shown to boost Runt-related transcription factor-2 and OPN protein expression [98].

### 12.11. Gum

Natural gum polysaccharides offer several benefits, including biodegradability, nontoxicity, and biocompatibility, which have led to their usage as prospective biomaterials in a range of biomedical applications. They are also superior to synthetic and semisynthetic polymers [277]. The usage of gums in the pharmaceutical sector and tissue engineering has increased dramatically in recent years. It is a top choice in scaffold synthesis due to its strong biocompatibility, biodegradability, and water solubility [278]. Natural gums offer great characteristics that allow them to be utilized in nanocomposites, including NPs and synthetic polymers for cell proliferation applications [279].

### 12.12. Heparin

One of the most water-soluble amphiphilic polysaccharides found is heparin. Certain evident advantages may be proven by altering the hydrophilic backbone of the heparin chain. Heparin may be used to make amphiphilic copolymers by adding certain hydrophobic chains. The intermolecular interaction between the hydrophilic component and the aqueous medium allows these copolymers to assemble into micellar-like NPs. The solubility of hydrophobic medicines is improved when they are coated with NPs, ensuring continual drug diffusion. The hydrophobic medicament binds to the heparin chain before condensing in the hydrophobic core, forming a heparin conjugate [280].

### 12.13. Chondroitin Sulfate

Chondroitin sulfate consists of the N-acetyl glucosamine and glucuronic acid. The repeating unit number of disaccharide might range between 40 and 100. Chondroitin sulfate is a good drug nanocarrier for the delivery of anthocyanin. By combining chondroitin sulfate with anthocyanin nanoparticles, the anthocyanin antioxidant activity may be preserved. According to studies, the nanoparticle stability of chondroitin sulfate–anthocyanin is improved eight times, and nanoparticles have considerable antitumor activity and better antioxidant in vitro than free anthocyanins [281]. Furthermore, the influence of lecithin on the chitosan features and chondroitin sulfate was studied, and the findings revealed that chitosan/chondroitin sulfate/lecithin nanoparticles had better qualities for the encapsulation of curcumin [282].

## 13. The Challenge and Future Direction of a New Generation of BTE Scaffold-Based Polysaccharide Hydrogel

As a result, the present biomaterial-based techniques for bone tissue creation and regeneration have significant limitations. The strong inflammatory responses caused by synthetic materials, which can lead to bacterial recontamination of tissues and inflammation as a result, are some of the most significant limitations [283]. These limitations stem primarily from the fact that current biomaterials for bone tissue engineering and regeneration lack specific temporal and spatial control over biologic signaling, which is required for progenitor cells to homing and differentiation in order to fully restore the tissue’s structural and functional characteristics [284]. Furthermore, most of the molecular knowledge of the regeneration effects achieved with existing biomaterials is lacking [285]. Finally, conventional polysaccharide-based biomaterials are mostly used to control infection and inflammation while stimulating the creation of reparative tissue [286]. To address these shortcomings of existing therapy, dental research has shifted its attention to developing and establishing more effective, dependable, and safe alternatives to standard scaffolds by developing novel regenerative techniques for renewing polysaccharide-based hydrogels [287].

A polysaccharide-based substance is used to create the hydrogel scaffolds. When exposed to the body’s regular biological processes, the materials break down spontaneously, making them typically safe and ecologically beneficial [288]. Bone ingrowth is aided by the porous design of scaffolds made of polysaccharide hydrogel. When employed as a culture substrate, bioscaffolds can increase cell adhesion, proliferation, differentiation, and tissue integration [289]. They may be made in a variety of ways, but the most prevalent include materials such as alginate [290], hyaluronic acid [291], and chitosan [292], as well as natural materials such as collagen/fibrinogen hydrogels. A number of new preparation techniques have been developed in order to manufacture predictable porous architecture hydrogels. An aqueous solution of polysaccharide hydroxyapatite (PHA) macromolecules generated through ionic cross-linking, for example, is presented as a new water-based hydrogel scaffold for bone tissue engineering [293,294,295]. A new generation of polysaccharide-based hydrogels has been developed. Biocompatibility, interconnected porosity, conductivity for attachment, capacity to stimulate proliferation and differentiation of committed cells, ability to assimilate inductive stimuli, adequate mechanical qualities, and biodegradability are all requirements [296,297]. Practically, a number of biomaterials have been created to meet these needs, the most essential of which is facilitating progenitor cell homing and differentiation by giving inductive signals for geographically and/or temporally directing tissue regeneration. Several novel materials, including extracellular matrix components, have been studied for this purpose (ECM), as well as other proteins, polysaccharides, peptides, natural or synthetic polymers, bioceramics, and recently, various innovative composites [298]. Each material has its own chemistry, composition, structure, and degradation profile, as well as the ability to be altered. As a result, the scaffold’s function has shifted from that of a passive carrier to that of a bioactive milieu with custom-tailored capabilities for repairing specific tissues [299]. As a result, polysaccharide hydrogels based on hydroxyapatite are one of the most promising choices for bone tissue scaffolds. The healing rate increases correspondingly when the microenvironment of a defect filler mimics the features of an injured site. In rabbit and minipig models, hydroxyapatite-based polysaccharide hydrogels have been shown to improve the healing of osteochondral lesions. Hydroxyapatite-based polysaccharide hydrogels can stimulate stem cell differentiation and increase cartilage and bone regeneration when employed as a medium for stem cell transport to the injury site [300,301,302]. Furthermore, injectable hydroxyapatite-based polysaccharide hydrogels successfully heal bone and cartilage lesions without the need for invasive surgical procedures [303]. However, hydroxyapatite-based polysaccharide hydrogels have certain drawbacks as a bone scaffold material, including their hydrophilic nature and low mechanical strength and integrity. Chemically modified and cross-linked hydroxyapatite-based polysaccharide hydrogels might be used to alleviate these disadvantages [304]. The porosity, mechanical strength, degradation rate, and stability of hydroxyapatite-based polysaccharide hydrogels might all be improved by cross-linking and altering them. The functional groups of hydroxyapatite-based polysaccharide hydrogels may be changed; hydroxyl groups can be changed by ester linkages, and carboxyl groups can be changed by hydrazide made using adipic dihydrazide [305]. Additionally, thiol and tyramine modification can be used to functionalize hydroxyapatite-based polysaccharide hydrogels [306]. The carboxyl groups of hydroxyapatite and the amine groups of tyramine form an amide link in tyramine-modified hydroxyapatite-based polysaccharide hydrogels [307]. Enzymatic degradation may be hampered by such chemical changes, which also vary chemical and mechanical characteristics. Various cross-linking approaches, such as radical polymerization and photo-cross-linking by chemical modification of hydroxyapatite-based polysaccharide hydrogels with methacrylate groups, might improve the mechanical characteristics of hydroxyapatite-based polysaccharide hydrogels. Chen et al. [308] revealed a potential strategy for the construction cross-linked hydrogel-based hydroxypropyl polysaccharide under the oxidation process for creating a new families of imine cross-linked hydrogels [309,310,311].

## 14. Conclusions

The field of natural polysaccharide polymers continues to captivate biomedical scientists and engineers. The most promising material to date for repairing bone defects is BTE scaffold. In BTE, synthetic biodegradable and natural polymers and composite materials can be used to create porous scaffolds. The scaffold hydrogel is a promising biomaterial in BTE due to its ability to carry several active compounds, such as steam cell, growth hormones, and inflammatory drugs. Natural bone stroma comprises collagen (inorganic material) and bone apatite (organic component). As a result, instead of a single material phase, the developed compound scaffolds incorporate both inorganic and organic phases. Because its chemical and crystalline traits are comparable to those of bone apatite, n-HA possesses superior biocompatibility, high plasticity, and excellent mechanical properties in the inorganic phase. Because of its ultrafine structure and huge surface area, which are both advantageous for cell–biomaterial interactions, n-HA has been extensively studied in the bone tissue engineering applications. On the other hand, for the organic phase, collagen is biodegradable, has excellent water retention ability, and is osteoinductive. Collagen can be employed as a synthetic bone graft substitute. However, the direct in vivo application of collagen in scaffolds is limited by its weak stiffness. The next generation compares the positive findings of different disciplines. Implementing goal-directed processing techniques to optimize the alternatives could be extremely effective and preserve resources. In summary, nano-inspired approaches can be used to create 3D polysaccharide hydrogel scaffold materials with superior performance, which are envisioned as the next generation of smart advanced materials. Practically, biomorphic transformation processes have the potential to provide useful and versatile tools for scientists working in fields other than biomedicine, that is, in fields where the chemical composition, structural hierarchy, and mechanical performance are combined and functionally important, for example, metamaterials, mechanics, photonics, optics, and energy. Natural structures with exceptional mechanical qualities connected to their 3D architecture, which reflect living models, might inspire a new generation of smart gadgets capable of hitherto unimaginable uses in the next decades.

## Data Availability

Not applicable.
